# The tomato chloroplast stromal proteome compendium elucidated by leveraging a plastid protein-localization prediction Atlas

**DOI:** 10.3389/fpls.2023.1020275

**Published:** 2023-08-28

**Authors:** Oindrila Bhattacharya, Irma Ortiz, Nathan Hendricks, Linda L. Walling

**Affiliations:** ^1^ Department of Botany and Plant Sciences, University of California, Riverside, Riverside, CA, United States; ^2^ Institute of Integrative Genome Biology, University of California, Riverside, Riverside, CA, United States

**Keywords:** chloroplast, leucine aminopeptidase, lumenal proteins, stroma, redox, protein homeostasis, proteomics, *Solanum lycopersicum*

## Abstract

Tomato (*Solanum lycopersicum*) is a model species for studying fruit development, wounding, herbivory, and pathogen attack. Despite tomato’s world-wide economic importance and the role of chloroplasts as metabolic hubs and integrators of environmental cues, little is known about the stromal proteome of tomato. Using a high-yielding protocol for chloroplast and stromal protein isolation, MudPIT nano-LC-MS/MS analyses, a robust in-house protein database (the Atlas) for predicting the plastid localization of tomato proteins, and rigorous selection criteria for inclusion/exclusion in the stromal proteome, we identified 1,278 proteins of the tomato stromal proteome. We provide one of the most robust stromal proteomes available to date with empirical evidence for 545 and 92 proteins not previously described for tomato plastids and the Arabidopsis stroma, respectively. The relative abundance of tomato stromal proteins was determined using the exponentially modified protein abundance index (emPAI). Comparison of the abundance of tomato and Arabidopsis stromal proteomes provided evidence for the species-specific nature of stromal protein homeostasis. The manual curation of the tomato stromal proteome classified proteins into ten functional categories resulting in an accessible compendium of tomato chloroplast proteins. After curation, only 91 proteins remained as unknown, uncharacterized or as enzymes with unknown functions. The curation of the tomato stromal proteins also indicated that tomato has a number of paralogous proteins, not present in Arabidopsis, which accumulated to different levels in chloroplasts. As some of these proteins function in key metabolic pathways or in perceiving or transmitting signals critical for plant adaptation to biotic and abiotic stress, these data suggest that tomato may modulate the bidirectional communication between chloroplasts and nuclei in a novel manner. The stromal proteome provides a fertile ground for future mechanistic studies in the field of tomato chloroplast-nuclear signaling and are foundational for our goal of elucidating the dynamics of the stromal proteome controlled by the solanaceous-specific, stromal, and wound-inducible leucine aminopeptidase A of tomato.

## Introduction

Plastids are essential organelles of green algae, land plants and some protists. Differentiating from proplastids, plastids develop into numerous forms, are tissue-specific and formed in response to endogenous signals (Jarvis and López-Juez, 2013). Well known for their role in photosynthesis, chloroplasts are metabolic hubs engaged in the biosynthesis of amino acids, starch, fatty acids, lipids, terpenoids, purine and pyrimidine bases, various pigments, vitamins, co-factors, as well as major biochemical pathways, such as nitrogen and sulfur metabolism (Rolland et al., 2012; Buchanan et al., 2015).

Approximately 2,500 proteins reside within chloroplasts (Abdallah et al., 2000). The vast majority are nuclear genome encoded, synthesized in the cytosol, imported into the chloroplast, and sorted into one of six sub-compartments (Cline and Dabney-Smith, 2008; Nakai, 2018; Thomson et al., 2020). N-terminal transit peptides facilitate the import of these proteins, while other proteins use non-canonical pathways for entering the chloroplast, including transit through the endoplasmic reticulum (Armbruster et al., 2009; Jarvis and López-Juez, 2013; Thomson et al., 2020).

Due to the emergence of its well-annotated genome in 2000 (Initiative, 2000), proteomes of *Arabidopsis thaliana* organelles including chloroplasts, mitochondria, peroxisomes, and vacuoles have been intensively studied (Carter et al., 2004; Kleffmann et al., 2004; Millar et al., 2006; Reumann et al., 2007; Zybailov et al., 2008). This includes the protein cohorts in Arabidopsis chloroplast sub-compartments: the envelope, stroma, thylakoid membrane, and lumen (Peltier et al., 2002; Schubert et al., 2002; Ferro et al., 2003; Friso et al., 2004; Peltier et al., 2006; Olinares et al., 2010). Several studies have combined gel or column fractionation in conjunction with mass spectroscopy (MS/MS) to elucidate the oligomeric complexes of the chloroplast (Peltier et al., 2006; Olinares et al., 2010; Lundquist et al., 2017). Finally, the proteomes of different plastid forms have also been established for developing plastids and chloroplasts from maize and eucalyptus (Majeran et al., 2012; Baldassi and Balbuena, 2022), potato leaf chloroplasts (Liu et al., 2022), wheat amyloplasts (Andon et al., 2002), rice and barley etioplasts (Von Zychlinski et al., 2005; Ploscher et al., 2011), tobacco proplastids (Baginsky et al., 2004), and chromoplasts from seven species (Siddique et al., 2006; Barsan et al., 2010; Barsan et al., 2012; Wang et al., 2013).

Of particular interest is the chloroplast’s role in sensing and transmitting signals to report organellar and cellular homeostasis (de Souza et al., 2017; Krupinska et al., 2020; Unal et al., 2020; Wang Y. et al., 2020). Chloroplasts have intimate and dynamic relationships with other organelles such as the nucleus, peroxisomes, mitochondria, and endomembrane system to enable signaling of cellular stress (Mehrshahi et al., 2013; Oikawa et al., 2019; Mullineaux et al., 2020). The diversity of signal pathways has primarily been elucidated genetically and biochemically in Arabidopsis allowing the discovery of a diverse set of metabolites (e.g., reactive oxygen species, isoprenoid intermediates, phosphonucleotides, chlorophyll precursors, carotenoid metabolites) and transcription factors to orchestrate these crucial communications (de Souza et al., 2017; Wang Y. et al., 2020). In addition, recent studies in Arabidopsis and other plants have shown that the chloroplast serves as a critical signaling hub in plant-pathogen interactions (Fernandez and Burch-Smith, 2019; Yang et al., 2021).

Defining the constituents of chloroplast proteomes and their dynamics in response to biotic and abiotic stress in crop plants is an emerging research area. In tomato, the stromal protein leucine aminopeptidase (LAP-A) controls expression of nuclear genes after herbivory, wounding and treatments with methyl jasmonate (Fowler et al., 2009; Scranton et al., 2013). The bifunctional LAP-A has both aminopeptidase and chaperone activities (Gu et al., 1999; Scranton et al., 2012) and LAP-A-dependent signal(s) may be generated post-translationally to orchestrate chloroplast-to-nucleus communication. With our long-term objective of understanding the LAP-A-dependent stromal proteome dynamics, we have determined a foundational component – tomato’s chloroplast stromal proteome.

Recent advances in sensitivity and accuracy in mass spectrometry joined with the availability of the annotated tomato nuclear and chloroplast genomes and a high-yielding tomato chloroplast and stromal protein isolation protocol, has allowed for an unprecedented in-depth understanding of tomato’s chloroplast stroma (Sato et al., 1999; Kahlau et al., 2006; Bhattacharya et al., 2020). Using nanoLC-MS/MS and two strategies to detect stromal proteins, we provide strong empirical evidence for 1,278 proteins in the tomato stromal proteome. With minimal contamination from other subcellular fractions of the chloroplast, this represents the largest stromal proteome to date and provides an important insight into the complexity of the eudicot stromal proteome. Our proteome adds 545 new proteins to previous studies that characterized the tomato chromoplast (Barsan et al., 2010; Barsan et al., 2012) and 130 proteins not previously identified in a wide range of *Arabidopsis thaliana* proteomics studies (Sun et al., 2009; Hooper et al., 2017). Tomato’s stromal proteins were manually curated and classified into ten protein functional categories allowing accessibility of our dataset.

## Materials and methods

### Chloroplast and stromal protein isolation

Tomato plants (*Solanum lycopersicum* UC82b) were grown to the three-to-four true-leaf stage (five-weeks-old) as described in Bhattacharya et al. (2020). Briefly, surface-sterilized tomato seeds were grown in UC Soil Mix 3 in flats with 18-section inserts in a growth chamber at 28 °C for 16 hr with 400 µmol m^-2^ s^-1^ light and 22 °C for 8 hr (dark). Plants were watered daily and fertilized weekly with a 0.35% (w/v) Miracle-Gro Tomato Plant Food solution. Twenty-seven hr prior to the chloroplast isolation, tomato plants were transferred to the dark to reduce starch. Five independent chloroplast preparations were made using leaves from 18 dark-adapted plants per preparation. Chloroplasts were isolated using a high-yielding chloroplast and stromal protein isolation method optimized for tomato leaves (Bhattacharya et al., 2020).

For each biological replicate, chloroplast soluble proteins (110 µg) were precipitated with four volumes of acetone for 16 hr at -20 °C and pelleted at 15,000 g for 30 min at 4 °C. The supernatant was discarded. The pellet was manually dislodged and washed with 1 mL of methanol to remove residual water. The sample was centrifuged at 15,000 g for 15 min at 4 °C. Supernatant was removed. The protein pellet was air-dried and stored at -20 °C until use.

To enhance identification of chloroplast stromal proteins, which may be obscured by abundant proteins in the 55- to 75-kDa range, stromal proteins (100 µg/lane) were fractionated by 12% SDS-PAGE and gels were stained with Coomassie Blue R-250 (Gu et al., 1996b; Rosenberg et al., 1997). The gel section with the 50- to 75-kDa proteins was excised and discarded. The proteins in remaining gel fragments were separated into three fractions based on mass (**Figure S1**). Proteins that were > 75-kDa (high mass) and < 20-kDa (low mass) proteins were pooled for analysis. The high plus low mass and the intermediate mass protein (50- to 20-kDa) samples had similar protein levels. Gel pieces were minced and destained in 50 mM ammonium bicarbonate in 50% acetonitrile with vigorous shaking at room temperature for 30 min. Destaining was repeated until gel pieces were devoid of Coomassie Blue R-250. After the final wash, gel pieces were dehydrated in 100% acetonitrile for 50 min at room temperature with vigorous shaking. Gel pieces were dried using a SpeedVac for 15 min at 30 °C and stored at -20 °C until use.

Acetone protein pellets were resuspended in 100 µL trypsin solution (10 µg/mL trypsin, 50 mM ammonium bicarbonate (pH 8), 10% acetonitrile) and incubated at 37 °C overnight. The gel protein samples were soaked with sufficient volume of trypsin solution (10 µg/ml trypsin, 50 mM ammonium bicarbonate) and incubated overnight at 37 °C. After trypsin digestion, five acetone-precipitated and three gel-extracted stromal protein samples were analyzed by nanoLC-MS/MS.

### NanoLC-MS/MS

A MudPIT approach was employed to analyze the trypsin-treated samples at the UC Riverside Institute of Integrative Biology Core by Dr. Songqin Pan. A nanoAcquity UPLC (Waters, Milford, MA) and an Orbitrap Fusion MS (Thermo Scientific, San Jose, CA) were configured to perform online 2D-nanoLC/MS/MS analysis. 2D-nanoLC was performed online using the nanoAcquity UPLC in an At-Column Dilution configuration. The first-dimension LC mobile phases were 20 mM ammonium formate (pH 10) (mobile phase A) and acetonitrile (mobile phase B) and was achieved with five-min elutions off a NanoEase trap column (Waters) using five stepwise increases in acetonitrile (13%, 18%, 21.5%, 27%, and 50% acetonitrile). A final flushing step with 80% acetonitrile was used to clean the column. Each fraction was then analyzed online using a second dimension LC gradient. The second dimension nano-UPLC method was described previously (Drakakaki et al., 2012).

Orbitrap Fusion MS method was based on a data-dependent acquisition (DDA) survey. The MS-acquired data from 1 to 69 min over a 70-min gradient. The nanoESI source was used with spray voltage at 2000 V, sweep gas at 0, and ion transfer tube temperature at 275 °C. Orbitrap mass analyzer was used for MS^1^ scan with resolution set at 60,000. MS mass range was 300-1800 m/z. AGC target for each scan was set at 500,000 with maximal ion injection time set at 100 ms.

Precursor ions with intensity 10,000 or higher were selected for MS^2^ scans, which were performed with the Ion-Trap mass analyzer in the rapid scan mode. The sequence of individual MS^2^ scans was from the most- to least-intense precursor ions using the top-speed mode and a cycle time of 4 sec. Precursor ions apex peak detection was enabled, using an expected peak width of 10 sec and Desired Apex Window set to 30%. The minimum peak intensity threshold was set to 1e4. Higher-energy collisional dissociation (HCD) with 25-35% normalized activation energy was used for fragmentation. The quadrupole was used for precursor isolation with 2 m/z isolation window. MS^2^ mass range was set to auto/normal with the first mass set at 120 m/z. Maximal injection time was 100 msec with the AGC target set at 10,000. Ions were injected for all available parallelizable time. A 120-sec exclusion window was applied to all abundant ions to avoid repetitive MS^2^ scanning on the same precursor ions using 10 ppm error tolerance. Charge states from 2 to 8 were selected for MS^2^ scan and undetermined charge states were excluded. All MS^2^ spectra were recorded in the centroid mode.

The raw MS files were processed and analyzed using Proteome Discoverer version 2.1 (Thermo Scientific, San Jose, CA). Sequest HT search engine was used to match all MS data to a tomato protein database (ITAG 2.4 annotation release) or the tomato Atlas (see below) and concatenated target/decoy databases were used for determining false discovery rates (Elias et al., 2005). The search parameters were the following: trypsin with two missed cleavages, minimal peptide length of six amino acids, MS^1^ mass tolerance 20 ppm, MS^2^ mass tolerance 0.6 Da, and Gln→pyro-Glu (N-term Q), oxidation (M), and N-terminal acetylation as variable modifications. Only proteins with 1% FDR cut-off were considered in the final result. Primary data is summarized in **Table S1**. The mass spectrometry proteomics data have been deposited to the ProteomeXchange Consortium via the PRIDE partner repository with the dataset identifier PXD035944.

### Annotation of the stromal proteome

All identified proteins (1% FDR) were manually annotated. Peptide spectral matches (PSMs) and frequency of detection in tomato eight stromal samples were the first criteria for inclusion/exclusion of the tomato chloroplast soluble proteome. Proteins that were detected once with 1 PSM, identified with a single peptide or sporadically identified (in less than 40% of the samples analyzed) were removed from consideration (Bhattacharya et al., 2020). The exceptions were proteins that had empirical evidence for residence within the chloroplast based on the tomato literature or Arabidopsis orthologs identified in the Plant Proteome Database (PPDB; http://ppdb.tc.cornell.edu/) (Sun et al., 2009), the Plastid Protein Database (plprot; http://www.plprot.ethz.ch/) (Kleffmann et al., 2006), and Subcellular Localization Database for Arabidopsis (SUBA4; http://suba.live/) (Hooper et al., 2017). The PPDB database was filtered for chloroplast-localized proteins with empirical evidence for localization within the chloroplast. The plprot database describes proteins localized in all plastid forms and was filtered for Arabidopsis homologs. SUBA4 was filtered for proteins with experimentally validated localizations within Arabidopsis plastids.

Proteins that were predicted to be chloroplast localized by more than two or more localization algorithms were also retained (see below). Gene names were based on the tomato literature, Sol Genomics database, updated with recent NCBI annotations, and, when appropriate, *Arabidopsis thaliana* orthologs, which were identified by the program Eggnog (http://eggnog5.embl.de/#/app/home) (Huerta-Cepas et al., 2019)(**Table S2**). Data from the primary literature and/or The Arabidopsis Information Resource site (TAIR; https://www.arabidopsis.org/) and Mercator and MapMan BIN ontologies (http://www.plabipd.de/portal/mercator-sequence-annotation/) were used for protein curation (Thimm et al., 2004; Lohse et al., 2014; Berardini et al., 2015). The full set of manually annotated proteins of the tomato stromal proteome is found in **Table S2A**. During manual annotation, we found that 63 genes/proteins were misannotated in the tomato genome (**Table S2B**).

### The tomato chloroplast protein Atlas

The 34,727 proteins of the deduced proteome of tomato (ITAG 2.4 annotation release) were downloaded from the Sol Genomics Network (http://www.solgenomics.net/) and imported into an R file, which included the amino acid sequences and gene annotations. Subcellular predictions for all deduced proteins were performed using four stand-alone software programs on the UCR Linux Biocluster, which included: TargetP version 1.1b (http://www.cbs.dtu.dk/services/TargetP/), ChloroP version 1.1 (http://www.cbs.dtu.dk/services/ChloroP/), WoLF PSORT version 2.0 (http://www.wolfpsort.org/), and YLoc (http://abi.inf.uni-tuebingen.de/Services/YLoc/webloc.cgi/) (Emanuelsson et al., 1999; Emanuelsson et al., 2000; Horton et al., 2007; Briesemeister et al., 2010). Subcellular predictions using the online version Predotar (http://urgi.versailles.inra.fr/predotar/predotar.html) were also made (Small et al., 2004). Proteins predicted to have a plastid location by one or more organellar prediction algorithms were included in the tomato chloroplast protein Atlas. Of the 87 conserved open-reading frames in the tomato chloroplast genome, six are in the inverted repeat and encode identical proteins; therefore, 81 chloroplast-genome encoded proteins were added to the Atlas (Daniell et al., 2006). The Atlas was maintained in an MS Excel file, with Sol Genomics Network (SGN) loci identifiers. In the absence of functional or experimental evidence from Arabidopsis databases or the literature, the reproducible detection and strong Atlas predictions were the criteria for retention of a protein in the tomato stromal proteome. As transmembrane domain algorithms often provide different predictions, TMpred (Hofmann and Stoffel, 1993), DeepTMHMM (Hallgren et al., 2022), and CCTOP (Dobson et al., 2015) were used to confirm the presence of transmembrane domains of tomato proteins. Lumenal transit peptides were predicted using PredSL (http://aias.biol.uoa.gr/PredSL/) and TargetP-2.0 (https://services.healthtech.dtu.dk/services/TargetP-2.0/) (Petsalaki et al., 2006; Almagro Armenteros et al., 2019). Venn diagrams were drawn using the VennDiagram package in RStudio Version 1.4.1717 open-source software (Chen and Boutros, 2011).

### Relative protein abundance

Relative protein abundance was calculated based on emPAI (exponentially modified protein abundance index) (Ishihama et al., 2005) using the acetone-precipitated protein data. PAI is the ratio of the number of detected proteins to the number of observable peptides per protein (Rappsilber et al., 2002) and was obtained for each protein from Thermo Scientific Proteome Discoverer (PD) 2.1 output. emPAI is calculated by PD as 10^PAI^ -1. The relative protein abundance (mol fraction) was calculated by dividing the emPAI of a protein by the sum of emPAIs of all the proteins in the entire dataset. The molar fraction was multiplied by 100 to obtain the mol % of each protein.

## Results

### Isolation and nanoLC-MS/MS analysis of the tomato chloroplast stromal proteome

A high-yielding chloroplast and stromal protein isolation protocol was used to identify the protein complement of the tomato chloroplast stromal proteome (Bhattacharya et al., 2020). Given the enhanced accuracy and sensitivity of the Orbitrap Fusion MS, we directly analyzed soluble chloroplast extracts that had chloroplast membranes removed by ultracentrifugation. A robust set of 2,325 proteins with a 1% FDR were obtained from the five biological replicates precipitated in 80% acetone and/or the three samples analyzed after 12% PAGE. The different methods of protein isolation were complementary. The acetone-precipitated and PAGE gel samples yielded 287 and 27 unique proteins, respectively (**Table S1**). Proteins were curated using a tomato chloroplast protein Atlas, databases with empirical evidence for a protein’s plastidial localization (plprot, SUBA4 and PPDB), relatedness to Arabidopsis orthologs, and evidence present in the literature (**Table S2**).

**Figure 1:**
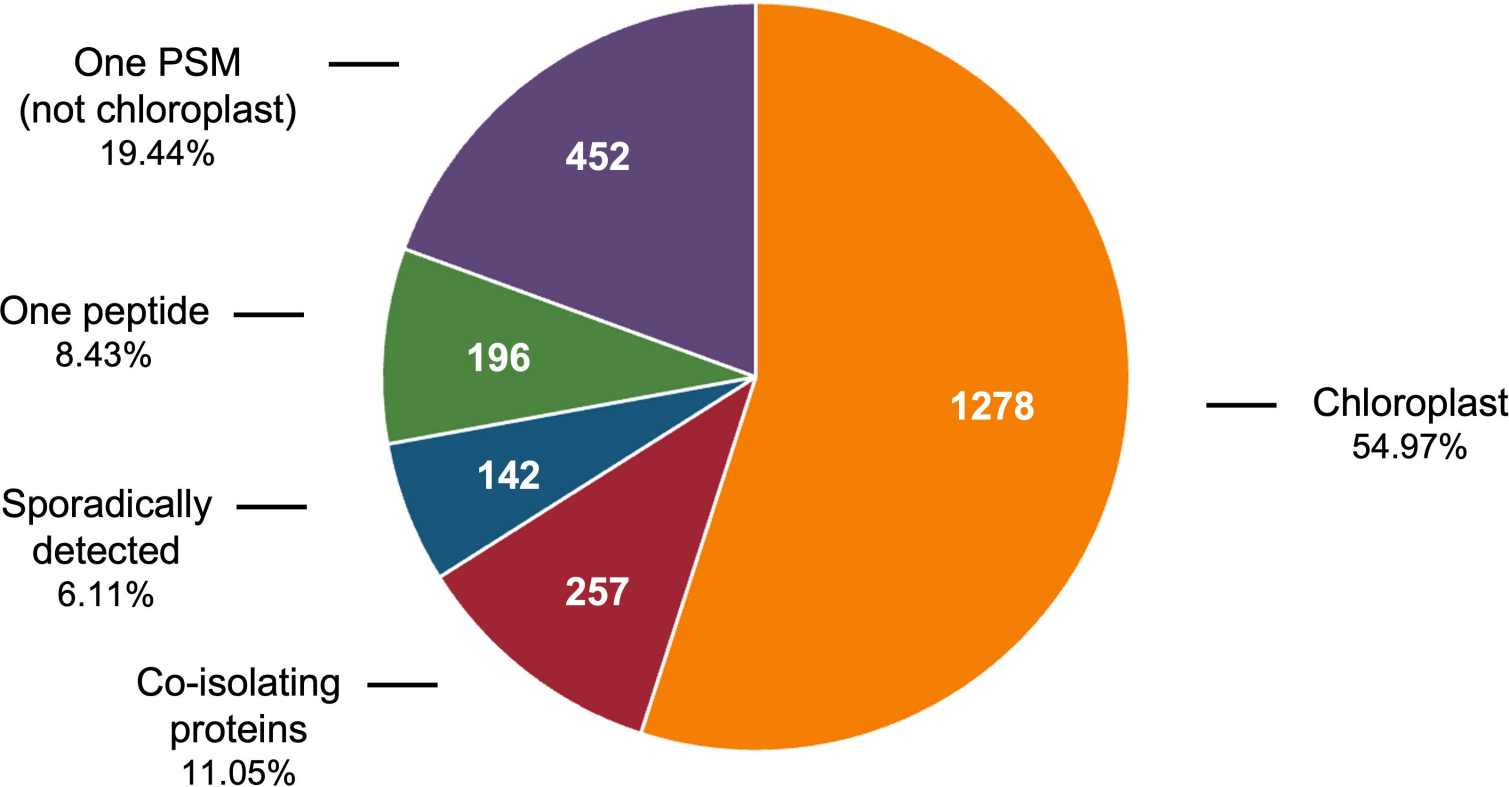
Classification of 1% FDR proteins identified in tomato chloroplast soluble extracts. The 2,325 proteins identified in the soluble extracts of tomato chloroplasts includes 1,278 chloroplast proteins, 257 co-isolating proteins (CIPs) that were reproducibly detected and 790 proteins that were removed from consideration because they were detected with one PSM, with one unique peptide, or sporadically (in less than 40% of the acetone or PAGE samples).

Rigorous criteria were used to define the tomato stromal proteome. Of the 2,325 proteins detected, 790 were removed from further analysis based on the fact that they were identified once by 1 peptide spectral match (PSM), with a single unique peptide, or sporadically (in less than 40% of the samples analyzed) (**Figure 1**). However, we retained any protein with a known chloroplast location to gain insights into low-abundance proteins in our stromal preparations. The remaining 1,535 proteins were unambiguously identified with 7,916 unique peptides and 60,830 peptide spectral matches (PSMs) from which 1,278 proteins were designated as the stromal proteome and 257 were classified as co-isolating proteins (CIPs), which were excluded from the stromal proteome (**Tables S1, S2**). CIPs were reproducibly isolated but their Arabidopsis homologs had empirical evidence for and/or protein localization algorithms strongly predicted residence in other subcellular compartments (Bhattacharya et al., 2020). CIPs may have dual localization within tomato cells; however, if chloroplast localized, CIPs do not use canonical transit peptides (Armbruster et al., 2009; Jarvis and López-Juez, 2013; Nakai, 2018; Thomson et al., 2020). It is also possible that the CIPs reflect the close proximity of and connections between other organelles such as the nucleus, peroxisome, mitochondria, and endomembrane system (Andersson et al., 2007; Islam and Takagi, 2010; Mehrshahi et al., 2013; Higa et al., 2014; Gao et al., 2016; Exposito-Rodriguez et al., 2017; Hooper et al., 2017; Barton et al., 2018; Oikawa et al., 2019; Mullineaux et al., 2020).

### Curation of the tomato stromal proteome: leveraging the tomato chloroplast protein Atlas and Arabidopsis protein localization databases

**Figure 2:**
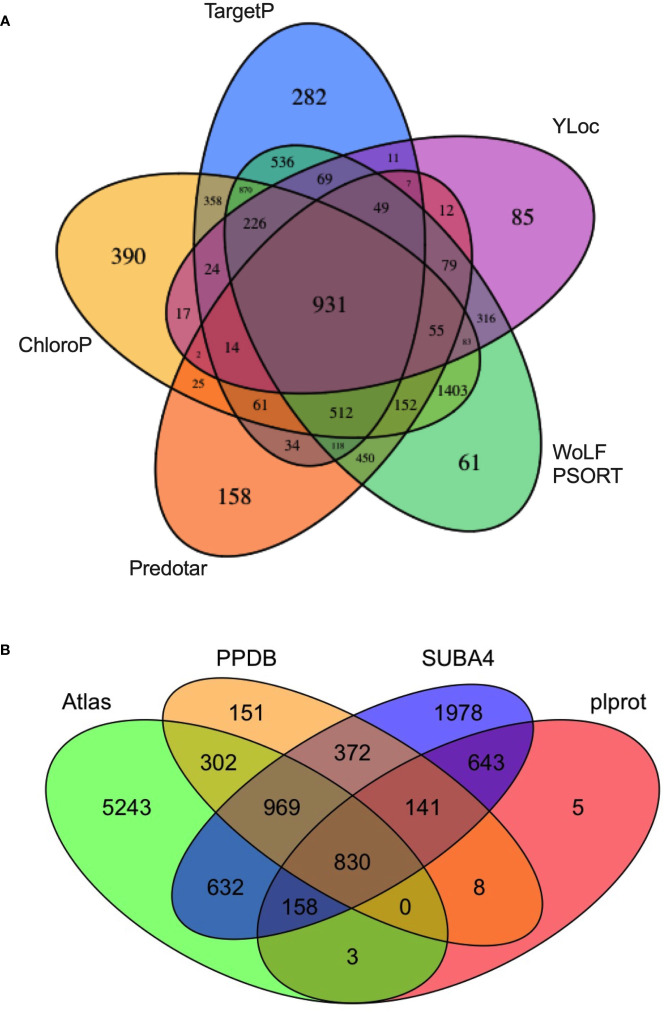
The tomato protein Atlas. **(A)** Source of proteins assigned to the tomato Atlas. A total of 7,473 nuclear-genome encoded proteins were predicted to be chloroplast localized by one or more of five subcellular localization programs: WoLF PSORT, Predotar, ChloroP, TargetP, and YLoc. The 81 plastid-genome encoded proteins, which are part of the Atlas, are not displayed. **(B)** A four-way Venn diagram compares the overlap of the tomato protein Atlas with *Arabidopsis thaliana* orthologs present in plprot, SUBA4, and PPDB. Proteins in the tomato chloroplast Atlas are found in **Table S3A**. Arabidopsis orthologs of predicted tomato chloroplast proteins were identified by Eggnog v5.0 and are found in **Table S3B**.

The use of multiple machine-learning algorithms is best practice for predicting the residence of plant proteins in subcellular compartments such as the chloroplast (Richly and Leister, 2004; Hooper et al., 2017). Here, five subcellular-localization programs (TargetP, ChloroP, Predotar, WoLF PSORT, and YLoc) were used to construct a theoretical tomato chloroplast proteome (the Atlas) (Emanuelsson et al., 1999; Emanuelsson et al., 2007; Horton et al., 2007; Briesemeister et al., 2010; Hooper et al., 2017) (**Table S3A**). The Atlas included 81 chloroplast genome-encoded proteins (Daniell et al., 2006; Kahlau et al., 2006) and 7,473 nuclear genome-encoded proteins predicted to be localized in the plastid by one or more programs (**Figure 2A**, **Table S3A**). The Atlas constitutes ~ 22% of the tomato genome making it a liberal predictor of chloroplast localization. This approach was reasonable since each algorithm brought different computational approaches to predict protein locations and was trained on different sets of proteins.

At the core of the Atlas are 931 proteins that were predicted to be chloroplast localized by all five programs (**Figure 2A**; **Table S3A**). No single algorithm identified all 1,278 proteins of the tomato stromal proteome and each algorithm identified a set of unique proteins ranging from 61 (WoLF PSORT) to 390 (ChloroP), stressing the contributions of each program to the Atlas (**Table S2**). Finally, based on the PPDB, plprot, and SUBA4 databases, only 2,903 of the proteins in the tomato Atlas (38.8%) had an Arabidopsis ortholog with empirical evidence for residence in the chloroplast (**Figure 2B**, **Table S3B**).

**Figure 3:**
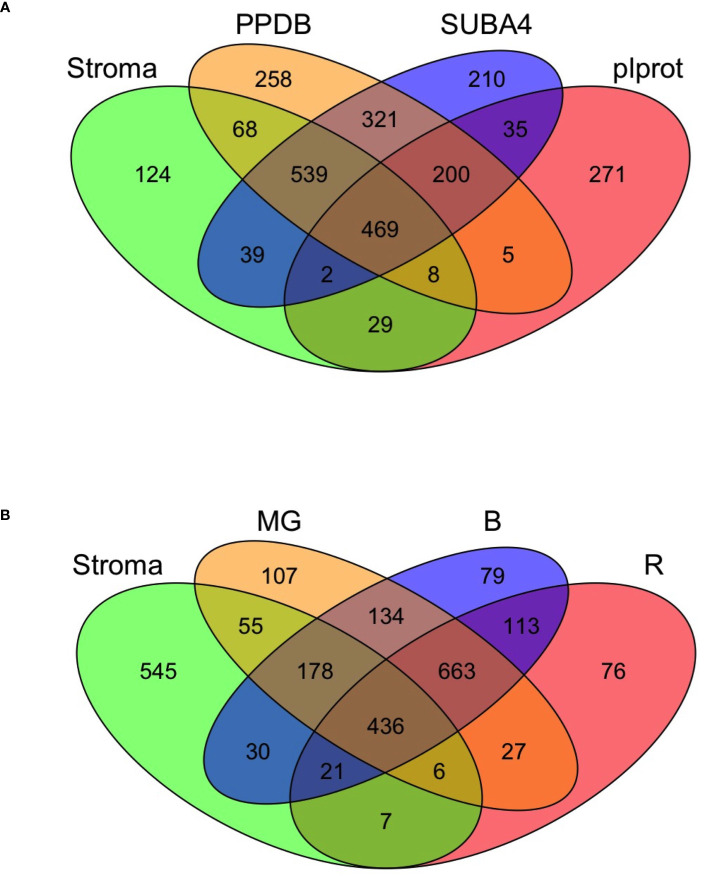
The tomato stromal proteome comparisons to Arabidopsis chloroplast proteins and tomato fruit proteins. **(A)** Comparison of the tomato stromal proteome with *Arabidopsis thaliana* chloroplast proteins present in the plprot, SUBA4 and PPDB databases. A core of 469 proteins with one or more Arabidopsis orthologs was detected in all three databases. Sixty proteins in the tomato stromal proteome had multiple Arabidopsis orthologs in all three databases, which brings the number of unique tomato proteins shared with the databases to 518 (**Tables S2, S3B**). **(B)** A four-way Venn diagram illustrates the overlap of the leaf stromal proteome with three tomato plastid proteomes from fruit in the mature green (MG), breaker and 10-d post breaker (B), and red (R) stages of fruit development.

Of the 1,278 proteins in the tomato stromal proteome, 89% were predicted by the Atlas and 84%, 88% and 43% of these proteins had one or more Arabidopsis homologs in PPDB, SUBA4 and plprot databases, respectively (**Tables S2, S3C**). A core of 469 proteins (36.7%) was detected in all three databases (**Table S2**; **Figure 3A**). These proteins were enriched for proteins involved in protein folding and targeting, tetrapyrrole synthesis, redox, and TCA metabolism; while proteins associated with DNA synthesis, amino acid metabolism, photosynthesis, and glycolysis were under represented.

### Sub-organellar localization of proteins and molar abundance

Immunoblots indicated that the tomato stromal proteome may harbor thylakoid lumenal proteins and should be depleted of thylakoid integral membrane proteins (Bhattacharya et al., 2020). TMpred, DeepTMHMM and CCTOP predicted 159 proteins with one or more transmembrane domains (Hofmann and Stoffel, 1993)(**Table S4A**). While 17 of these proteins had an unknown location within the chloroplast, proteins associated with the thylakoid membrane (95), the envelope (30), both chloroplast membrane systems (3), and plastoglobules (3) were detected. Based on these numbers, membrane proteins constituted 12.4% of the stromal proteome. However, it should be noted that 46 of the membrane proteins were sporadically detected (<40% of acetone or gel samples), making estimated percentage of membrane proteins 8.8% (**Table S4A**).

Fifty-nine proteins that reside within the tomato chloroplast lumen were identified and represented 4.6% of the stromal proteome (**Table 1**). The lumenal proteins had a diverse array of functions including 12 immunophilins (cyclophilins and FKPBs), three C-terminal processing proteases, three DEG protease subunits, 11 lumenal proteins associated with the PSI, PSII, CytB6/f, and NAD(P)H complexes, as well as 22 proteins involved photosystem maintenance or assembly (**Table 1**). We detected nine of the ten tomato FKBP proteins predicted to be within the chloroplast (Waseem et al., 2018); only FKBP12, which was predicted to be localized to both the cytosol and chloroplast, was not detected. We also detected eight lumenal proteins with orthologs in Arabidopsis that were not detected in earlier studies (**Table S4B**), as well as tomato’s PPO-F and PPO-A (Newman et al., 1993).

**Table 1:** Tomato lumenal proteins^A^.

Functional Class	Gene and protein descriptors				Proteomics				
	Tomato Gene ID	Protein Name^B^	Descriptor ^C^	Arabidopsis homolog	Peptides	PSMs	emPAI	Mol %	# times detected
**Defense**	Solyc08g074620	PPO-E	Polyphenol oxidaseE	–	31	853	233.62	1.5065	6
	Solyc08g074680	PPO-A	Polyphenol oxidaseA	–	20	115	1.89	0.0122	5
**Proteolysis**	Solyc12g097030	CTPA2	Carboxyl-terminal processing protease 2	At4g17740	18	76	3.76	0.0242	8
	Solyc02g071190	CTPA1	Carboxyl-terminal-processing protease 1	At5g46390	14	48	5.06	0.0326	8
	Solyc03g059260	CTPA3	Carboxyl-terminal-processing protease 3	At3g57680	2	4	0.39	0.0025	4
	Solyc02g086830	DEG1	DegP Protease 1	At3g27925	13	137	11.92	0.0768	8
	Solyc08g048550	DEG5	DegP Protease 5	At4g18370	4	29	2.42	0.0156	8
	Solyc02g067360	DEG8	DegP Protease 8	At5g39830	9	30	3.44	0.0222	8
**Protein** **folding**	Solyc08g006540	FKBP13	Peptidyl-prolyl *cis-trans* isomerase	At5g45680	6	38	2.83	0.0183	8
	Solyc04g054520	FKBP16-2	Peptidyl-prolyl *cis-trans* isomerase	At4g39710	4	7	4.18	0.0269	7
	Solyc04g015040	FKBP16-3	Peptidyl-prolyl *cis-trans* isomerase	At2g43560	6	93	15.68	0.1011	8
	Solyc09g008650	FKBP16-4	Peptidyl-prolyl *cis-trans* isomerase	At3g10060	1	1	0.21	0.0014	1
	Solyc02g069130	FKBP17-1	Peptidyl-prolyl *cis-trans* isomerase	At4g19830	3	8	1.68	0.0109	8
	Solyc03g119150	FKBP17-3	Peptidyl-prolyl *cis-trans* isomerase	At1g18170	2	2	0.67	0.0043	5
	Solyc04g082660	FKBP18	Peptidyl-prolyl *cis-trans* isomerase	At1g20810	3	7	0.78	0.0050	6
	Solyc11g033284	FKBP19^A^	Peptidyl-prolyl *cis-trans* isomerase	At5g13410	3	19	6.20	0.0400	7
	Solyc10g039270	FKBP20-2	Peptidyl-prolyl *cis-trans* isomerase	At3g60370	5	13	1.15	0.0074	7
	Solyc02g086910	CYP38	Peptidyl-prolyl *cis-trans* isomerase	At3g01480	13	194	19.69	0.1270	8
	Solyc01g009990	CYP20-2 (PNSL5)	Peptidyl-prolyl *cis-trans* isomerase	At5g13120	10	191	30.62	0.1975	8
	Solyc12g013580	CYP37	Peptidyl-prolyl *cis-trans* isomerase	At3g15520	7	30	1.68	0.0109	8
**Cytb6/f complex**	Solyc12g005630	PETC	Cytochrome b6-f complex iron-sulfur subunit	At4g03280	3	44	1.61	0.0104	8
	Solyc04g082010	PETE	Plastocyanin	At1g20340	1	44	5.31	0.0342	8
	Solyc02g068930	PETJ	Cytochrome c6	At5g45040	2	10	0.43	0.0027	7
**NAD(P)H complex**	Solyc10g054420	PNSL1	Photosynthetic NDH subunit of lumenal location 1	At2g39470	3	7	1.15	0.0074	7
	Solyc05g007780	PNSL2	Photosynthetic NDH subunit of lumenal location 2	At1g14150	1	6	0.78	0.0050	5
	Solyc10g006530	PNSL3	Photosynthetic NDH subunit of lumenal location 3	At3g01440	2	4	1.51	0.0098	5
**Photosystem I**	Solyc08g013670	PSAN	Photosystem I reaction center subunit N	At5g64040	1	16	0.33	0.0022	7
**Photosystem II**	Solyc02g065400	PSBO-2 (OEE1)	PSII oxygen-evolving enhancer protein 1	At3g50820	15	300	15.68	0.1011	8
	Solyc02g090030	PSBO-2 (OEE1)	PSII oxygen-evolving enhancer protein 1	At3g50820	15	309	13.13	0.0846	8
	Solyc07g044860	PSBP (OEE2)	PSII oxygen-evolving enhancer protein 2	At1g06680	13	484	35.52	0.2290	8
	Solyc02g079950	PSBQ (OEE3)	PSII oxygen-evolving enhancer protein 3	At4g05180	18	199	99.00	0.6384	8
**Photosystem assembly, stability, repair and unknown functions**	Solyc12g005180	LCNP	Lipocalin in the plastid	At3g47860	8	37	6.02	0.0388	8
	Solyc02g014150	HCF136	Photosystem II stability/assembly factor HCF136	At5g23120	12	223	12.11	0.0781	8
	Solyc02g083270	LTO1(VKOR)	Lumen thiol oxidoreductase 1 (Vitamin K epoxide reductase)	At4g35760	3	5	1.15	0.0074	2
	Solyc01g106090	PPD1	PsbP domain protein, PPD1, PSB27-like	At4g15510	11	68	6.08	0.0392	8
	Solyc04g009420	PPD2	PS II oxygen evolving complex protein PPD2	At2g28605	9	29	9.00	0.0580	8
	Solyc12g094720	PPD3	PS II oxygen evolving complex protein PPD3	At1g76450	7	92	9.00	0.0580	8
	Solyc04g064670	PPD4	PsbP domain-containing protein 4	At1g77090	7	46	7.11	0.0459	8
	Solyc08g067840	PPD5	PsbP domain-containing protein 5	At5g11450	6	23	1.89	0.0122	8
	Solyc06g065490	PPD6	PsbP domain-containing protein 6	At3g56650	4	68	2.51	0.0162	8
	Solyc03g114930	PPL1	PS II reaction center PsbP family protein	At3g55330	7	49	5.58	0.0360	8
	Solyc07g054290	PSB27-H1	PS II repair protein	At1g03600	3	9	1.51	0.0098	8
	Solyc09g076030	PSB27-H2	PS II repair protein	At1g05385	8	37	4.88	0.0315	8
	Solyc09g064500	PSB28	PS II reaction center Psb28 protein	At4g28660	4	21	3.22	0.0207	6
	Solyc06g076480	TL15-1	Thylakoid lumen 15.0-kDa protein	At2g44920	4	27	1.15	0.0074	8
	Solyc10g084040	TL15-2	Thylakoid lumen 15.0-kDa protein	At5g52970	7	44	7.11	0.0459	8
	Solyc12g009600	TL16.5 (MPH2)	Thylakoid lumenal 16.5-kDa protein	At4g02530	11	85	10.94	0.0705	8
	Solyc03g082890	TL17.4	Thylakoid lumenal 17.4-kDa protein	At5g53490	7	39	3.92	0.0253	8
	Solyc03g019660	TL17.9	Thylakoid lumenal 17.9-kDa protein	At4g24930	6	29	6.50	0.0419	7
	Solyc01g098640	TL18.3	Thylakoid lumenal 18.3-kDa protein	At1g54780	10	34	5.31	0.0342	8
	Solyc01g087040	TL19	Thylakoid lumenal 19-kDa protein	At3g63540	9	122	315.23	2.0328	8
	Solyc08g079110	TL20.3	Thylakoid lumenal 20.3-kDa protein	At1g12250	9	74	6.36	0.0410	8
	Solyc04g074640	TL29	Thylakoid lumenal 29-kDa protein	At4g09010	8	34	2.59	0.0167	8
**Xanthophyll synthesis**	Solyc04g051610	VDE-like	Violaxanthin de-epoxidase-related protein	At2g21860	1	1	0.17	0.0011	1
	Solyc04g050930	VDE1	Violaxanthin de-epoxidase	At1g08550	17	98	5.58	0.0360	8
**unknown**	Solyc05g012600	–	Unknown Protein	At2g03420	1	4	0.26	0.0017	4
	Solyc09g005740	–	Chloroplast lumen common family protein	At2g37400	7	30	1.68	0.0109	8
	Solyc12g019550	–	Unknown Protein	At1g21500^D^	2	4	2.16	0.0139	3

^A^ Tomato lumenal proteins were identified based on empirical evidence (PPDB) or based on lumenal localization predicted by both PredSL and TargetP version 2. One protein was inferred by putative function (VDE1-like). The SolGenomics ID for FKB19 (in ITAG1.2) was changed to Solyc11g033284.1.1 (ITAG4); see **Table S2C**. Complete information about the lumenal proteins are found in **Table S2A** or **Table S4B**.

^B^ Names of tomato proteins were based on the literature (reference provided) and NCBI annotation (identified in BlastP searches). In a small number of cases, tomato protein names were assigned based on NCBI annotations and the Arabidopsis orthologs.

^C^ Some Sol Genomics descriptors were updated when NCBI annotations and Arabidopsis gene annotations were aligned.

^D^ Solyc12g019550 has similarity to the hypothetical protein At1g21500, which is predicted is lumenal (Peltier et al., 2002; Schubert et al., 2002).

**Figure 4:**
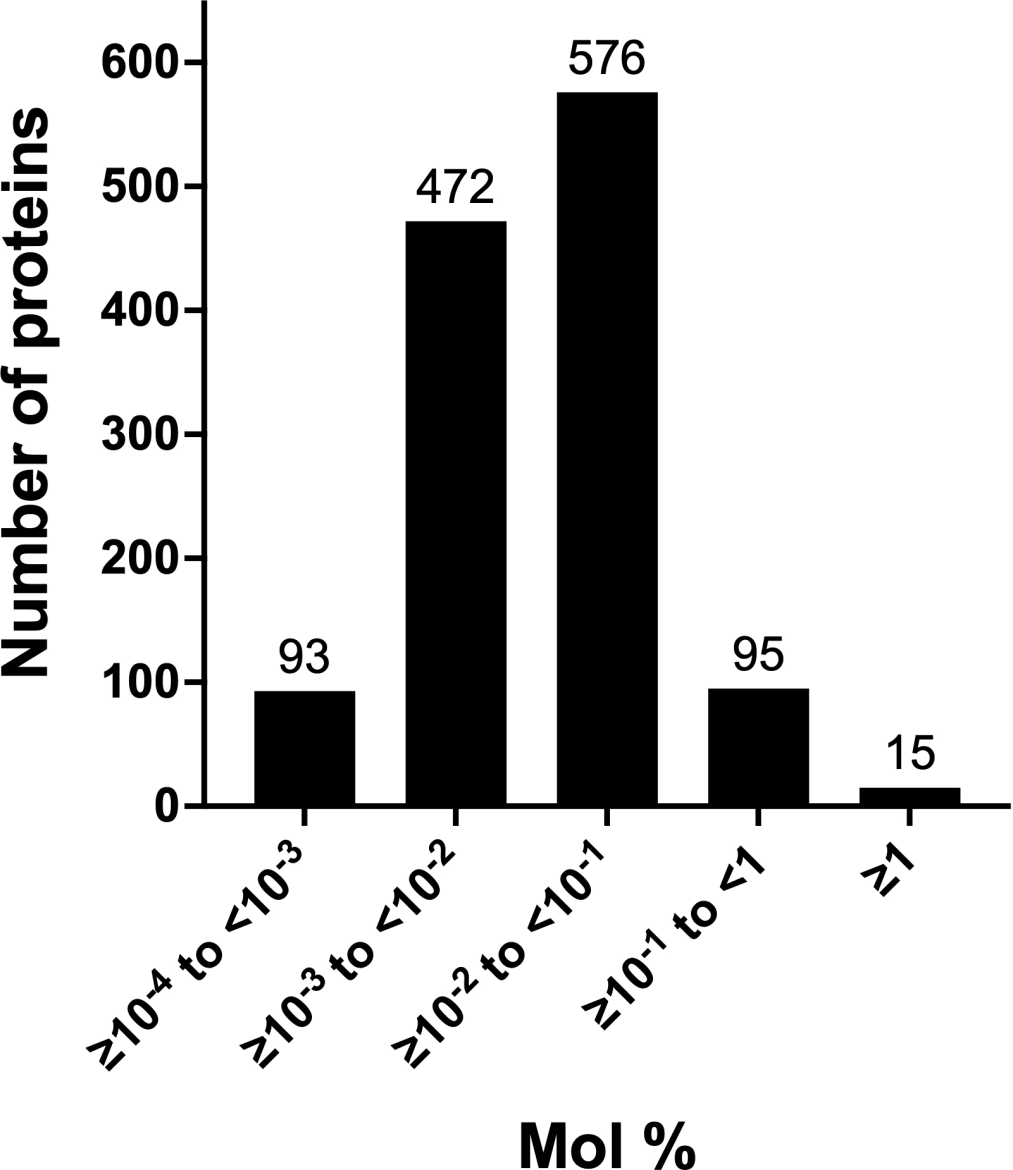
Abundance classes of leaf stromal proteins. The abundance of the 1,251 acetone-precipitated proteins of the leaf stromal proteome was determined by calculating the emPAI and mol % of the proteome. Five protein classes were defined by their relative abundance. The numbers of proteins in each emPAI class are provided above the bar.

The total number of chloroplast membrane and lumenal proteins overestimated their contribution to the stromal proteome (17.1%). A better assessment was provided by the exponentially modified protein abundance index (emPAI) (**Table S2**). We used the emPAI to normalize the abundance of stromal proteins in acetone-precipitated samples. emPAI is based on the number of detected peptides versus the number of observable peptides per protein to provide an estimate of a protein’s molar abundance (Ishihama et al., 2005). The mol % of tomato’s stromal proteins varied over a 5.7 x 10^4^-fold range, with the majority of proteins in the 10^-3^ to 10^-2^ mol % categories (**Figure 4**; **Table S2**). Membrane proteins represented a 1.9 mol % of the stromal proteome (**Table S4A**), while the 59 lumenal proteins accounted for 5.8 mol % (**Table 1**). The most abundant lumenal protein was TL19, constituting 33% of the lumen protein mass. Collectively, tomato chloroplast membrane and lumenal proteins constituted 7.7% of the mass of proteins in the stromal proteome, representing a minor proportion of the tomato stromal proteome. These data strongly support previous immunoblot data indicating low levels of proteins from other compartments of the chloroplast (Bhattacharya et al., 2020).

### Relative abundance of proteins and novel proteins in the tomato chloroplast stromal proteome

A small number of studies have provided insights into eudicot stromal proteomes. To elucidate chloroplast complexes and soluble proteomes in Arabidopsis, these studies used SDS-PAGE (Ferro et al., 2003; Peltier et al., 2006), size exclusion chromatography (Peltier et al., 2006), affinity chromatography (Bayer et al., 2011), or blue native-PAGE (Lundquist et al., 2017) to prefractionate proteins prior to MS analyses. To benchmark the tomato stromal proteome relative to the Arabidopsis stromal proteome, we compared the relative abundance of the tomato stromal proteins to the relative normalized abundance of the 241 Arabidopsis stromal proteins identified by Peltier et al. (2006).

The top two classes of tomato stromal proteins had mol % values ranging from > 0.1 to 13.9 (**Figure 4**; **Table 2**). The rankings of these 110 proteins were compared to their Arabidopsis orthologs (Peltier et al., 2006). Although of varying abundance and rankings, 19 of the 23 most abundant proteins in Arabidopsis were detected in tomato’s top two abundance classes (**Table S4C**); the other abundant Arabidopsis proteins were detected but at lower levels (**Table S4C**). For the most abundant tomato stromal proteins, there were 26 proteins with two or more orthologs in tomato relative to a single protein in Arabidopsis (**Table 2**). For eleven, both orthologous proteins of tomato were in the top two abundance classes including: RuBisCo activase2 (RCA2A, RCA2B), chaperone DnaK (HSC70-2a, HSC70-2b), Clp protease subunit C (CLPC1, CLPC2), elongation factor Tu (EFTuA, EFTuB), fructose-bisphosphate aldolase (FBA1, FBA2), glycolate oxidase1 (GLO1A, GLO1B), glyceraldehyde-3-phosphate hydrogenase (GAPB1, GAPB2), ketol-acid reductoisomerase (At3g58610-like1 and 2), 29-kDa ribonucleoprotein (CP29A, CP29B), superoxidase dismutase (Fe-SOD2A, Fe-SOD2B), and triosephosphate isomerase (pdTPI1, pdTPI2). For the remaining sixteen, the two orthologous proteins accumulated to different levels suggesting different mechanisms of regulation. Forty-seven proteins in the top-two protein cohorts were not detected by Peltier et al. (2006) (**Table 2**).

**Table 2:** Top 110 tomato stromal proteins.

Tomato Gene ID^A^	Protein name^B^	Gene product^C^	Ranking (Mol %)	Mol %	Arabidopis homolog^D,E^	Arabidopsis abundance class^F^
544163620	ATPB	ATP synthase CF1 beta subunit	1	13.886	Atcg00480	4
544163621	RBCL	RuBisCo large subunit	2	10.412	Atcg00490	1
544163653	RPS19	Ribosomal protein S19	3	6.442	Atcg00820	4
Solyc06g073260	CSP41B	41-kDa chloroplast stem-loop binding protein	4	4.477	At1g09340	3
544163671	RPS15	Ribosomal protein S15	5	4.062	Atcg01120	4
Solyc06g007760	YCF54	Ycf54 protein, Low chlorophyll accumulation (LCAA)	6	4.062	At5g58250	nd
Solyc01g087040	TL19	Thylakoid lumenal 19-kDa protein	7	2.033	At3g63540	nd
Solyc10g076350	–	Macrophage migration inhibitory factor family protein	8	2.033	At5g01650	nd
Solyc08g074630	PPO-F^K^	Polyphenol oxidase F	9	1.948	–	–
Solyc08g074620	PPO-E	Polyphenol oxidase E	10	1.507	–	–
Solyc06g071790	EF-TuB	Elongation factor TuB	11	1.335	At4g20360	2
Solyc10g086150	CP29B^H^	29-kDa RNA-binding protein B, HopU1 effector target	12	1.202	At2g37220	4
Solyc10g086580	RCA2A^G^	RuBisCo activase	13	1.169	At2g39730	2
Solyc09g007850	CP29A^H^	29-kDa RNA-binding protein A	14	1.069	At2g37220	4
Solyc03g095180	Fe-SOD2^L^	Superoxide dismutase	15	1.016	At4g25100 (At5g51100)	3
544163678	RPL23	Ribosomal protein L23	16	0.940	Atcg01300	nd
Solyc02g083500	AANH-like	Adenine nucleotide alpha hydrolase family protein	17	0.853	At5g66090	3
Solyc03g112150	EF-TuA	Elongation factor TuA	18	0.797	At4g20360	2
Solyc09g065180	CSP41A	41-kD chloroplast stem-loop binding protein	19	0.738	At3g63140	3
Solyc09g011080	RCA2B^G^	RuBisCo activase	20	0.710	At2g39730	2
544163619	ATPE	ATP synthase CF1 epsilon subunit	21	0.638	Atcg00470	nd
Solyc02g079950	OEE3	Oxygen-evolving enhancer protein 3, PsbQ	22	0.638	At4g21280 (At4g05180)	4
Solyc02g086740	RPL12-A^O^	50S ribosomal protein L12-A	23	0.638	At3g27830	nd
544163598	ATPA	ATP synthase CF1 alpha subunit	24	0.506	Atcg00120	nd
Solyc04g007010	KIROLA-like^P^	KIROLA-like, Major latex-like protein 43-like	25	0.433	At1g70890	nd
Solyc12g056830	ATPD	ATP synthase delta subunit	26	0.433	At4g09650	nd
Solyc12g042060	CLPC1^N^	Clp protease subunit CLPC1	27	0.408	At5g50920	3
Solyc01g080280	GS2	Glutamine synthetase	28	0.400	At5g35630	1
Solyc02g086730	RPL12-C	50S ribosomal protein L12-C	29	0.400	At3g27840	nd
Solyc12g013810	TRX-m4.1^J^ (Trx-m1/4)	Thioredoxin m	30	0.400	At1g03680 (At3g15360)	4
Solyc01g057830	RPS1A	30S ribosomal protein S1	31	0.393	At5g30510	nd
Solyc01g106430	PPA6	Inorganic pyrophosphatase family protein	32	0.387	At5g09650	3
Solyc01g103450	HSC70-2^I^	Chloroplast heat shock protein 70	33	0.375	At5g49910	4
Solyc07g066610	cpPGK1	Phosphoglycerate kinase1	34	0.343	At1g56190	2
Solyc07g062060	MSRB1	Peptide methionine sulfoxide reductase B	35	0.328	At1g53670	nd
Solyc02g020940	GAPA-2	Glyceraldehyde-3-phosphate dehydrogenase	36	0.317	At1g12900	2
Solyc02g086820	CA1	Carbonic anhydrase 1, SA-binding protein 3	37	0.317	At3g01500	2
Solyc08g006070	AIG2-like	AIG2-like protein	38	0.293	At4g31310	nd
Solyc10g018300	TKL1	Transketolase 1	39	0.293	At3g60750	2
Solyc03g118240	CHLM	Magnesium-protoporphyrin IX methyltransferase	40	0.280	At4g25080	nd
Solyc02g083810	LFRN^R^ PETH	Ferredoxin–NADP reductase	41	0.275	At1g20020	3
Solyc02g084440	FBA3^M^	Fructose-bisphosphate aldolase	42	0.270	At4g38970	2
Solyc08g076220	PRK	Phosphoribulokinase/uridine kinase	43	0.265	At1g32060	3
544163637	CLPP1	Clp protease proteolytic subunit P1	44	0.250	Atcg00670	3
Solyc02g080540	ATPC	ATP synthase gamma chain	45	0.250	At4g04640	nd
Solyc04g009030	GAPA-1	Glyceraldehyde-3-phosphate dehydrogenase subunit 2	46	0.250	At3g26650	2
Solyc11g066390	SOD3	Superoxide dismutase	47	0.250	At2g28190	nd
Solyc07g056540	GLO1	Glycolate oxidase 1	48	0.238	At3g14420	nd
Solyc07g044860	OEE2	Oxygen-evolving enhancer protein 2, PsbP	49	0.229	At1g06680	2
Solyc11g069790	CPN60A2	Chaperonin - RuBisCo LS binding protein (A	50	0.228	At2g28000	3
Solyc04g074750	CP33C	Polyadenylate-binding protein 1-A	51	0.225	At4g09040	nd
Solyc12g010840	-	Ketol-acid reductoisomerase	52	0.222	At3g58610	3
Solyc01g097460	RPI3	Ribose-5-phosphate isomerase	53	0.216	At3g04790	nd
544163595	RPS16	Ribosomal protein S16	54	0.197	Atcg00050	nd
Solyc01g009990	CYP20-2 (PNSL5)	Peptidyl-prolyl *cis-trans* isomerase, cyclophilin-type	55	0.197	At5g13120	nd
Solyc04g008710	-	Glutamic acid-rich protein-like	56	0.197	At3g24506	nd
Solyc05g005480	-	NADPH-dependent alkenal/one oxidoreductase	57	0.197	At1g23740	3
Solyc05g009030	IMDH	3-isopropylmalate dehydrogenase	58	0.197	At1g31180	4
Solyc05g052710	RPS31 (PSRP4)	30S ribosomal protein S31	59	0.197	At2g38140	nd
Solyc08g006780	STIC2-like	Suppressor of Tic40-2	60	0.197	At4g30620	nd
Solyc08g081570	MeCPS	2-C-methyl-D-erythritol 2 4-cyclodiphosphate synthase	61	0.197	At1g63970	nd
Solyc08g079180	EF-G	Elongation factor G	62	0.179	At1g62750	3
Solyc01g100520	CLPP5	Clp protease proteolytic subunit P5	63	0.177	At1g02560	3
Solyc03g121910	TS1	Threonine synthase	64	0.177	At4g29840	nd
Solyc01g108600	PREP1	Presequence protease	65	0.174	At1g49630	nd
Solyc03g007110	CLPT1	Clp protease T1 subunit	66	0.173	At4g25370	5
Solyc06g048410	Fe-SOD2 (PAP9)	Superoxide dismutase	67	0.167	At5g51100	nd
Solyc03g118340	CLPC2^N^	Clp protease C2 subunit	68	0.151	At5g50920	3
Solyc12g010380	AK5	Adenylate kinase-like protein	69	0.151	At5g35170	nd
Solyc06g048730	UROD2 (HEME2)	Uroporphyrinogen decarboxylase	70	0.146	At2g40490	nd
Solyc05g005880	RPS13	30S ribosomal protein S13	71	0.143	At5g14320	nd
Solyc01g079790	APL1	Glucose-1-phosphate adenylyltransferase	72	0.140	At5g19220	5
Solyc03g063560	GLU1	Ferredoxin-dependent glutamate synthase	73	0.134	At5g04140	3
Solyc01g009080	-	FHA domain containing protein	74	0.132	At2g21530	nd
Solyc01g111120	pdTPI-2A	Plastid triosephosphate isomerase	75	0.132	At2g21170	2
Solyc05g052600	SBPase	Sedoheptulose-1,7-bisphosphatase	76	0.132	At3g55800	2
Solyc07g025520	-	Methyltransferase type 11	77	0.132	At4g29590	nd
Solyc07g053280	-	Ketol-acid reductoisomerase	78	0.132	At3g58610	3
Solyc07g063190	Trx-m4.3	Thioredoxin	79	0.132	At3g15360	nd
Solyc11g006020	NDHO	NAD(P)H-quinone oxidoreductase subunit O	80	0.132	At1g74880	nd
Solyc12g094640	GAPB^S^	Glyceraldehyde-3-phosphate dehydrogenase B	81	0.132	At1g42970	2
Solyc02g086910	CYP38	Peptidyl-prolyl *cis-trans* isomerase cyclophilin-type	82	0.127	At3g01480	3
Solyc04g009200	GSA	Glutamate-1-semialdehyde-2 1-aminomutase	83	0.127	At5g63570	4
Solyc01g028810	CPN60B2	Chaperonin, RuBisCo LS-binding protein	84	0.127	At3g13470	3
Solyc01g110360	FBA1^M^	Fructose-bisphosphate aldolase	85	0.122	At4g38970	2
Solyc02g085100	-	Putative glucose-6-phosphate 1-epimerase	86	0.122	At5g66530	4
Solyc03g111840	-	28-kDa ribonucleoprotein	87	0.122	At4g24770	3
Solyc03g120430	GLYK	Glycerate kinase	88	0.122	At1g80380	nd
Solyc04g082630	GAPB	Glyceraldehyde-3-phosphate dehydrogenase B	89	0.121	At1g42970	2
Solyc06g053600	At1g04420-like1	Oxidoreductase aldo/keto reductase family protein	90	0.120	At1g04420	4
Solyc01g006980	MCAT	Malonyl CoA-acyl carrier protein transacylase	91	0.118	At2g30200	4
Solyc01g108630	NIR	Nitrite reductase	92	0.118	At2g15620	4
Solyc03g120850	CPN60B1	Chaperonin - RuBisCo LS binding protein	93	0.118	At1g55490	3
Solyc06g076790	-	Uncharacterized protein	94	0.118	At3g47070	nd
Solyc09g008670	OMR1A	Threonine dehydratase 2	95	0.116	At3g10050	nd
Solyc02g062340	FBA2^M^	Fructose-bisphosphate aldolase	96	0.116	At4g38970	2
544163615	RPS4	Ribosomal protein S4	97	0.114	Atcg00380	2
Solyc02g088610	CLPB3	ATP-dependent chaperone ClpB	98	0.111	At5g15450	4
Solyc01g105060	-	Thioesterase superfamily protein	99	0.108	At5g10160	nd
Solyc10g054870	pdTPI-2B	Triosephosphate isomerase	100	0.108	At2g21170	2
Solyc12g010020	LapA1	Leucyl aminopeptidase A1	101	0.108	At4g30920-like	nd
Solyc12g089210	OTC2	Ornithine carbamoyltransferase	102	0.108	At1g75330	4
Solyc12g094430	GSTF5	Glutathione S-transferase	103	0.108	At2g30860	nd
Solyc01g005520	MET1	Tetratricopeptide TPR2 repeat protein	104	0.105	At1g55480	nd
Solyc01g006560	LOXF TomLoxF	Lipoxygenase	105	0.105	At3g45140	3
Solyc03g111610	-	HAD-superfamily hydrolase subfamily protein	106	0.104	At3g48420	3
Solyc02g065400	PSBO-1 OEE1-1	PS II Oxygen-evolving enhancer protein 1	107	0.101	At3g50820	3
Solyc04g007790	KIROLA-like	KIROLA-like protein, Major latex-like protein	108	0.101	At1g70890	nd
Solyc04g015040	FKBP16-3	Peptidyl-prolyl *cis-trans* isomerase	109	0.101	At2g43560	nd
Solyc06g072470	RPL29	50S ribosomal protein L29	110	0.101	At5g65220	nd

^A^ Tomato gene IDs are from Sol Genomics.

^B^ Names of tomato genes were curated from the literature, Sol Genomics database, NCBI and/or were guided by names of Arabidopsis orthologs. See **Table S2** for NCBI accessions and literature citations. Several genes had two or three paralogs in tomato versus a single gene in Arabidopsis.

^C^ Identities of tomato proteins were confirmed by reciprocal BLASTP searches for the tomato and Arabidopsis homologs at NCBI and Sol Genomics.

^D^ Some tomato proteins do not have orthologs in Arabidopsis. These proteins are designated with a dash (-).

^E^ For proteins in multigene families, the closest Arabidopsis ortholog is provided. However, there were cases when an Arabidopsis ortholog was not detected by Peltier et al. (2006). In these cases, the next mostly closely-related homolog (name in parentheses) was identified using BlastP searches and its corresponding rank provided.

^F^ Peltier et al. (2006) classified 241 proteins into concentration ranking groups 1 (most abundant) and 4 (least abundant). When the Arabidopsis homolog of a tomato protein was not detected it is indicated by “nd”.

^G^ There are two tomato RuBisCo activase proteins similar to At2g39730 (AtRCA2) in tomato (Solyc10g086580 -RCA2A and Solyc09g011080-RCA2B). A third tomato RCA protein (RCA1) is similar to At1g73110 (AtRCA1).

^H^ There are two 29-kDa RNA-binding proteins (A and B) in tomato. In Arabidopsis, these proteins are also a HopU1 effector target.

^I^ There are two chloroplast *Hsc70-2* genes in tomato (Solyc01g103450 and Solyc11g020040) that are more similar to the Arabidopsis Hsc70-2 (At5g49910) than Hsc70-1 (At4g24280). Phylogenetic analysis of the tomato Hsc70 protein family was performed by Vu et al. (2019), but gene names were not assigned.

^J^ *TRX-m* gene family is expanded relative to Arabidopsis. The TRX-m nomenclature is based on reciprocal BLAST-P searches of tomato TRX-m and Arabidopsis TRX-m proteins and names were based on relatedness and phylogenetic trees of homologs. TRX-m4 was previously designated as TRX-m1/4 (Cheng et al., 2014). Current phylogenic trees unambiguously classify this protein as a TRX-m4. Gene family names are found in **Table S2** and **Table S10** (protein folding).

^K^ *PPO* gene nomenclature was previously established by Newman et al. (1993).

^L^ The tomato *SOD* gene family is expanded relative to Arabidopsis. While Arabidopsis has one *Fe-SOD2* gene, there are two *Fe-SOD2* genes in tomato. SOD proteins detected in tomato’s stromal proteome are found in the Redox Table (**Table S9D**).

^M^ *FBA* gene nomenclature was based on Cai et al. (2016).

^N^ In our hands, there are two tomato CLPC1 proteins with greatest protein identity to Arabidopsis’ CLPC1 (At5g0920) and a weaker identity to AtCLPC2 (At3g11830). This differs from the analyses of D’Andreas et al. (2018); despite this, we have used the D’Andreas et al. CLPC nomenclature.

^O^ There are two *RPL12* genes in tomato. Names are based on Sol Genomics designations. Solyc02g086740 encodes RPL12-A and Solyc02g086730 encodes RPL12-C.

^P^ Four major latex proteins (MLPs) were identified in the tomato stromal proteome (**Table S2**). NCBI designates them as KIROLA or KIROLA-like and we have retained this nomenclature.

^Q^ Based on reciprocal BLASTP searches there is only one *PETE* gene in tomato, while there are two in Arabidopsis. The tomato PETE is mostly closely related to ATPETE2.

^R^ There are two leaf ferredoxin NADP reductases (LFNR, PETH) in tomato (Solyc02g083810, Solyc02g062130) that are similar to the AtLFNR2 (AtFNR2, At1g20020) and AtLFNR1 (FNR1, AT5G66190).

^S^ Based on reciprocal BLASTP searches there are two GAPB paralogs in tomato, while there is one in Arabidopsis (At1g42970).

Reciprocally, of the 23 most abundant Arabidopsis stromal proteins, all but one (a ROC4-like protein with no tomato ortholog) were detected in the tomato stromal proteome but their relative rankings (by mol %) were significantly different (**Table S4C**) (Peltier et al., 2006). While the RuBisCo large subunit (RBCL) was one of the most abundant proteins in both studies, there was a striking difference in the abundance of the RuBisCo small subunits. Peltier et al. (2006) reported the abundance of an RBCS protein pool, which ranked 2 in abundance. In contrast, the analogous tomato RBCS pool had a combined mol % of 0.228, which ranked the pool as 51 in the tomato stromal proteome (**Table S4C**). Furthermore, some tomato proteins, such as 2-CYS-Prx1, 2-CYS-Prx2, CPN20, and LOX2, were not even in the top 110 most-abundant proteins of the tomato stromal proteome. Collectively, these data indicate the mechanisms that dictate stromal protein abundance are significantly different in these plant species.

Comparisons of tomato stromal proteome with Arabidopsis chloroplast proteins catalogued in PPDB, SUBA4 or plprot showed that 130 stromal proteins were not previously detected in plastids (**Table 3**). A majority (72%) of the novel proteins were reproducibly detected (in >40% of acetone and/or gel samples) and 82.4% of the novel proteins were predicted to reside within the chloroplasts by two or more algorithms (**Table 3**, **Table S2A**). The abundance of the novel stromal proteins ranged from 1.95 mol % to < 3.8 x 10^-4^ mol % and totaled 4.96 mol % of the stromal proteome. Strikingly, six defense-associated proteins (LAP-A1, LAP-A2, PPOE, PPOF, AIG2-like, and KIROLA) were abundant and, collectively, accounted for 81% of the mass of the novel proteins based on mol %. Most novel stromal proteins were not abundant and were likely identified due to the enhanced sensitivity, accuracy and resolution of the Orbitrap Fusion MS.

**Table 3:** Proteins present in the tomato stromal proteome but not reported by PPDB, SUBA4 or plprot^A^.

Classification	Tomato Gene ID^B^	Protein name^C^	Protein Descriptor^D^	Arabidopsis homolog
**DNA binding: transcription factors & histones**	Solyc03g120840	TINY-like	Ethylene-responsive transcription factor (TINY-like)	At5g11590
	Solyc06g074780	H2B.1-like	Histone H2B.1-like	At5g59910
	Solyc01g079110	Histone H3.2-like	Histone H3 variant	At4g40030
	Solyc04g081150	Histone H3.2-like	Histone H3 variant	At4g40030
	Solyc08g061140	OCP3	Over-expression of cationic peroxidase	At5g11270
	Solyc02g072260	–	SAP-like protein BP-73	–
	Solyc05g010070	–	Zinc finger family protein, Zim17-type	At1g68730
**DNA binding: ssDNA binding & helicases**	Solyc05g014690	RECQ-like	ATP-dependent DNA helicase	At1g27880
	Solyc07g053690	–	OB-fold DNA binding domain protein	At4g28440
	Solyc10g081050	–	Phage-related exonuclease	At1g67660
**RNA biogenesis**	Solyc03g121260	–	23S rRNA (Uracil-5-)-methyltransferase, RumA	At3g21300
	Solyc09g015930	–	ATP-dependent RNA helicase	At3g58570
	Solyc12g096510	CREF	Chloroplast RNA editing factor	At5g06540
	Solyc02g079210	MORF1	Multiple organellar RNA editing factor 1	At4g20020
	Solyc05g054960	MORF5	Multiple organellar RNA editing factor 5	At1g32580
	Solyc10g062340	–	Polyadenylate-binding protein	At2g44710
	Solyc10g047000	RAE1	RNA export factor 1	At1g80670
	Solyc01g086900	–	RNA methyltransferase TrmH group 2	At5g26880
	Solyc02g088540	–	tRNA guanosine-2’-O-methyltransferase	At5g15390
	Solyc03g118680	VAR3-like	Zinc finger protein, RanBP2-type	At1g48570
**Protein synthesis**	Solyc08g062920	EF-2	Elongation factor EF-2	At1g56070
	Solyc02g055440	YCED2	Large rRNA subunit accumulation protein, YCED homolog 2	At3g19800
	Solyc12g096390	PTRHD1	Putative peptidyl-tRNA hydrolase	At5g10700
	Solyc07g062930	PRMA-like	Ribosomal protein L11 methyltransferase-like protein	At5g53920
	Solyc05g005800	ThrRS	Threonyl-tRNA synthetase	At5g26830
	Solyc09g007540	ValRS1	Valyl-tRNA synthetase	At1g14610
**Protein homeostasis**	Solyc03g005340	AARE1	Acyl-amino acid releasing enzyme	At4g14570
	Solyc03g097130	–	ATP-binding protein (kinase)	At5g16810
	Solyc08g008170	CDPK19	Calcium dependent protein kinase 19	At4g23650
	Solyc09g015360	DJC73	Co-chaperone protein DnaJ	At5g59610
	Solyc12g056850	DJC65	Co-chaperone protein DnaJ	At1g77930
	Solyc06g076020	HSC70-1	Heat shock protein 70	At5g02490
	Solyc12g010020	LapA1	Leucyl aminopeptidase (acidic)	–
	Solyc12g010030	LapA2	Leucyl aminopeptidase (acidic)	–
	Solyc10g008020	–	Methyltransferase	–
	Solyc06g084030	–	Methyltransferase like 7	At1g69523
	Solyc02g069130	FKBP17-1	Peptidyl-prolyl *cis-trans* isomerase, FKBP-type	At4g19830
	Solyc11g044310	PAP1	Proline iminopeptidase	At2g14260
	Solyc01g005380	–	SET domain containing protein	At1g24610
	Solyc08g081200	TIC32-like	Short-chain dehydrogenase TIC 32	At4g23420
	Solyc07g026950	XPD	Xaa-Pro dipeptidase	At4g29490
**Redox**	Solyc08g059760	APX6	L-ascorbate peroxidase 6	At4g32320
	Solyc12g056230	GPXle2	Glutathione peroxidase	At4g11600
	Solyc08g080940	GPXle1	Glutathione peroxidase-like encoding 1	At4g11600
	Solyc07g020860	TPX1	Thioredoxin dependent peroxidase	At1g65980
**Biotic & abiotic stress**	Solyc08g006070	AIG2-like	AIG2-like protein	At4g31310
	Solyc04g005700	KIROLA	KIROLA	At5g28010
	Solyc08g023660	KIROLA-like	Major latex-like protein (KIROLA-like)	At1g70840
	Solyc08g074630	PPO-F	Polyphenol oxidase	–
	Solyc08g074680	PPO-A	Polyphenol oxidase	–
	Solyc08g074620	PPO-E	Polyphenol oxidase	–
	Solyc10g006760	–	Universal stress protein	At1g11360
**Amino acid biosynthesis**	Solyc12g010180	ASB2	Anthranilate synthase beta subunit	At1g24807
	Solyc01g098550	TSA	Tryptophan synthase alpha chain	At3g54640
	Solyc04g051860	SK1	Shikimate kinase	At2g21940
**CHO metabolism & glycolysis**	Solyc01g073740	CSY4	Citrate synthase	At2g44350
	Solyc06g071920	GAPC-1 (cytosol)	Glyceraldehyde-3-phosphate dehydrogenase	At3g04120
	Solyc05g014470	GAPC-2 (cytosol)	Glyceraldehyde 3-phosphate dehydrogenase	At1g13440
	Solyc02g086610	ICDH	Isocitrate dehydrogenase-like protein	At1g65930
	Solyc09g064240	–	Kinase pfkB family protein	At4g28706
	Solyc11g069040	–	Lactoylglutathione lyase	At1g08110
	Solyc10g005400	MIOX	Myo-inositol oxygenase	At1g14520
	Solyc03g114250	–	Phosphoglycerate mutase family protein	At5g62840
	Solyc04g005160	PGD2	6-phosphogluconate dehydrogenase	At3g02360
	Solyc08g081390	PGM-like	Phosphoglycerate mutase-like protein	At3g05170
	Solyc02g077680	PHS2	Phosphorylase	At3g46970
	Solyc02g091340	–	Pyridoxal kinase isoform 1, pyridoxal kinase	At5g37850
**Lipid metabolism**	Solyc01g067730	ACP5	Acyl carrier protein	At3g05020
	Solyc12g006870	–	Acyl-protein thioesterase 2	At5g20060
	Solyc06g064640	ABHD11	Alpha/beta hydrolase	At4g10030
	Solyc07g008310	CMO-like	Choline monooxygenase-like	At4g29890
	Solyc11g072640	–	*trans*-2-enoyl-CoA reductase	At1g49670
**Nucleotide metabolism**	Solyc05g052260	–	Appr-1-p processing domain protein	At2g40600
	Solyc04g080430	–	5’-nucleotidase	At1g75210
	Solyc10g037900	–	Dihydroorotate dehydrogenase	At2g44760
	Solyc01g089970	NDK1	Nucleoside diphosphate kinase	At4g09320
	Solyc08g082430	NDK3	Nucleoside diphosphate kinase	At4g23895
	Solyc02g080780	–	Orotidine 5’-phosphate decarboxylase	At1g62250
	Solyc04g039620	PRS3	Ribose-phosphate pyrophosphokinase 3	At1g10700
**Secondary metabolism**	Solyc05g056540	ADH1B	Alcohol dehydrogenase-like protein	At5g63620
	Solyc08g014360	CAD6	Cinnamyl alcohol dehydrogenase-like protein	At4g39330
	Solyc01g105890	TPS5 (MTS1)	Linalool synthase	At3g25810
	Solyc06g005720	–	Tropinone-reductase-like39	At2g29260
**Miscellaneous**	Solyc04g073990	ANN1	Annexin	At1g35720
	Solyc03g115110	–	ATP synthase gamma chain	At2g33040
	Solyc04g007550	–	ATP synthase subunit beta	At5g08680
	Solyc02g086880	FNADH1	Formate dehydrogenase	At5g14780
	Solyc02g063070	GRF7	14-3-3 protein beta_alpha-1	At5g16050
	Solyc08g014480	–	Lactase-like protein	At3g54440
	Solyc01g090670	–	Nuclear pore glycoprotein p62	At2g45000
	Solyc12g035650	NUP54	Nucleoporin p54	At1g24310
	Solyc02g080220	PME	Pectinesterase	At1g11580
	Solyc05g050530	PPOX2	Pyridoxamine 5’-phosphate oxidase family protein	At2g46580
	Solyc07g066580	STR1	Mercaptopyruvate sulfurtransferase-like protein	At1g79230
**Uncharacterized functions**	Solyc05g012370	–	Alpha/beta fold family protein	At1g13820
	Solyc02g086080	–	Alpha/beta hydrolase fold	At5g38360
	Solyc01g080140	–	Alpha-beta hydrolase super family	At5g19050
	Solyc08g013840	SGPP	Broad-range sugar phosphate phosphatase	At2g38740
	Solyc03g019680	CBS (CBSPB5-like)	CBS domain containing protein	At5g50640
	Solyc05g043430	–	Carboxymethylenebutenolidase-like protein	At2g32520
	Solyc02g094430	ELT5	Esterase/lipase/thioesterase family protein	At5g41130
	Solyc06g060880	–	FAD/NAD(P)-binding oxidoreductase family protein	At2g29720
	Solyc07g066280	–	Methyltransferase domain protien	At5g64150
	Solyc02g093550	–	Methyltransferase type 11	At3g01660
	Solyc03g114660	–	Pentatricopeptide repeat-containing protein	At3g57430
	Solyc01g111470	–	Pentatricopeptide repeat-containing protein	At2g16880
	Solyc03g083280	–	Pentatricopeptide repeat-containing protein	At3g49240
	Solyc03g098440	–	Small glutamine-rich tetratricopeptide repeat-containing protein A	At3g17670
	Solyc08g006830	–	S-adenosyl-L-methionine-dependent methyltransferase	At3g62000
	Solyc03g118860	–	UDP-N-acetylglucosamine–N-acetylmuramyl-(pentapeptide) pyrophosphoryl-undecaprenol N-acetylglucosamine transferase	At1g73740
	Solyc02g070800	YCF23	Uncharacterized Ycf23 protein	–
	Solyc09g006000	–	Zinc/iron-chelating domain protein	At5g02710
**Unknown**	Solyc03g044630	–	Conserved domain protein	At2g41120
	Solyc07g063510	–	Low-quality, uncharacterized protein	–
	Solyc08g074450	–	Protein of unknown function DUF1997	At4g31115
	Solyc01g068470	–	Uncharacterized protein	At2g38780
	Solyc02g068350	–	Uncharacterized protein	–
	Solyc04g072400	–	Uncharacterized protein	At1g36320
	Solyc05g007680	–	Uncharacterized protein	At1g26761
	Solyc05g055550	–	Uncharacterized protein	At3g10405
	Solyc09g074950	–	Uncharacterized protein	At4g02480
	Solyc10g005830		Uncharacterized protein	At2g35820
	Solyc10g081280	–	Uncharacterized protein	–
	Solyc01g096400	–	Unknown Protein	–
	Solyc02g063300	–	Unknown Protein	At5g38060
	Solyc04g074770	–	Unknown Protein	–
	Solyc05g024330	–	Unknown Protein	–
	Solyc06g042980	–	Uncharacterized protein	At3g12590
	Solyc12g056350	–	Unknown Protein	At2g32500
	Solyc02g076950	–	UPF0052 domain protein	At2g34090

^A^ All primary data for the novel proteins are found in **Table S2A**. An expanded version of **Table 3** is found in **Table S2C**. **Table S2C** includes # of unique peptides, # Psms, # peptides, emPAI, mol %, and Atlas predictors of protein localization.

^B^ Gene IDs are from the Sol Genomics database or NCBI. While not reported in the Arabidopsis databases, the ortholog of the Solyc03g097130 protein was detected by Bayer et al. (2011) after affinity purification of ATP- and metal-binding proteins.

^C^ Names of tomato proteins were based on the literature and NCBI annotation (identified in BlastP searches). In a small number of cases, tomato protein names were assigned based on NCBI annotations and the Arabidopsis homolog. In ITAG2.4, the three *Lap* genes of tomato are misannotated (*LapA1*, *LapA2* and *LapN*). This is being resolved in ITAG4.0. The LAP-A1 and LAP-A2 proteins are only discriminated in the COOH portion of their proteins, luckily these were present in the ITAG2.1 gene designators. The new loci will be Solyc12g10020 (*LapA1*), Solyc12g10030 (*LapA2*), and Solyc12g10040 (*LapN*). LAP-A proteins are not present in Arabidopsis; At4g30920 is the ortholog to tomato *LAP-N*. See **Table S2** for additional information.

^D^ Some Sol Genomics descriptors were updated with NCBI or Arabidopsis gene annotations based on the literature or reciprocal BLASTP data.

Forty-two of the novel proteins had roles in RNA biogenesis, protein biogenesis, redox, or stress responses, ten were transcription factors or DNA-binding proteins, and 32 proteins had roles in cellular metabolism spanning amino acid to secondary metabolism (**Table 3**). Unknown proteins and proteins with uncharacterized functions dominated, representing 28% of the novel proteins. Finally, 14 proteins did not have orthologs in Arabidopsis including: two leucine aminopeptidases (LAP-A1, LAP-A2) (Gu et al., 1996a), three tomato polyphenol oxidases (PPO-F, PPO-E, and PPO-A) (Newman et al., 1993; Tran et al., 2012), YCF23, and a methyltransferase.

### Functional comparisons of the tomato leaf stromal and fruit plastid proteomes

While the proteomes of tomato fruit are well-characterized (Sant’Ana and Lefsrud, 2018), few studies have focused on the plastids of tomato fruit or leaves (Barsan et al., 2010; Barsan et al., 2012; Tamburino et al., 2017). Barsan et al. (2010; 2012) identified 1,932 proteins in plastids undergoing the chloroplast to chromoplast transition associated with fruit ripening (**Table S3D**). A core of 436 proteins were shared with our leaf stromal proteome and the proteomes of mature-green, breaker and red fruit plastids with reflecting shared housekeeping and biochemical functions. In addition, 545 proteins unique to the leaf stromal proteome were identified (**Figure 3B**; **Table S3**). Of the 81 chloroplast-genome encoded proteins, 44 were detected in the leaf stromal proteome (**Table S5A**). Collectively the leaf stromal and fruit plastid proteomes provided empirical evidence for 55 of the chloroplast-genome encoded proteins (**Tables S3D**).

**Figure 5:**
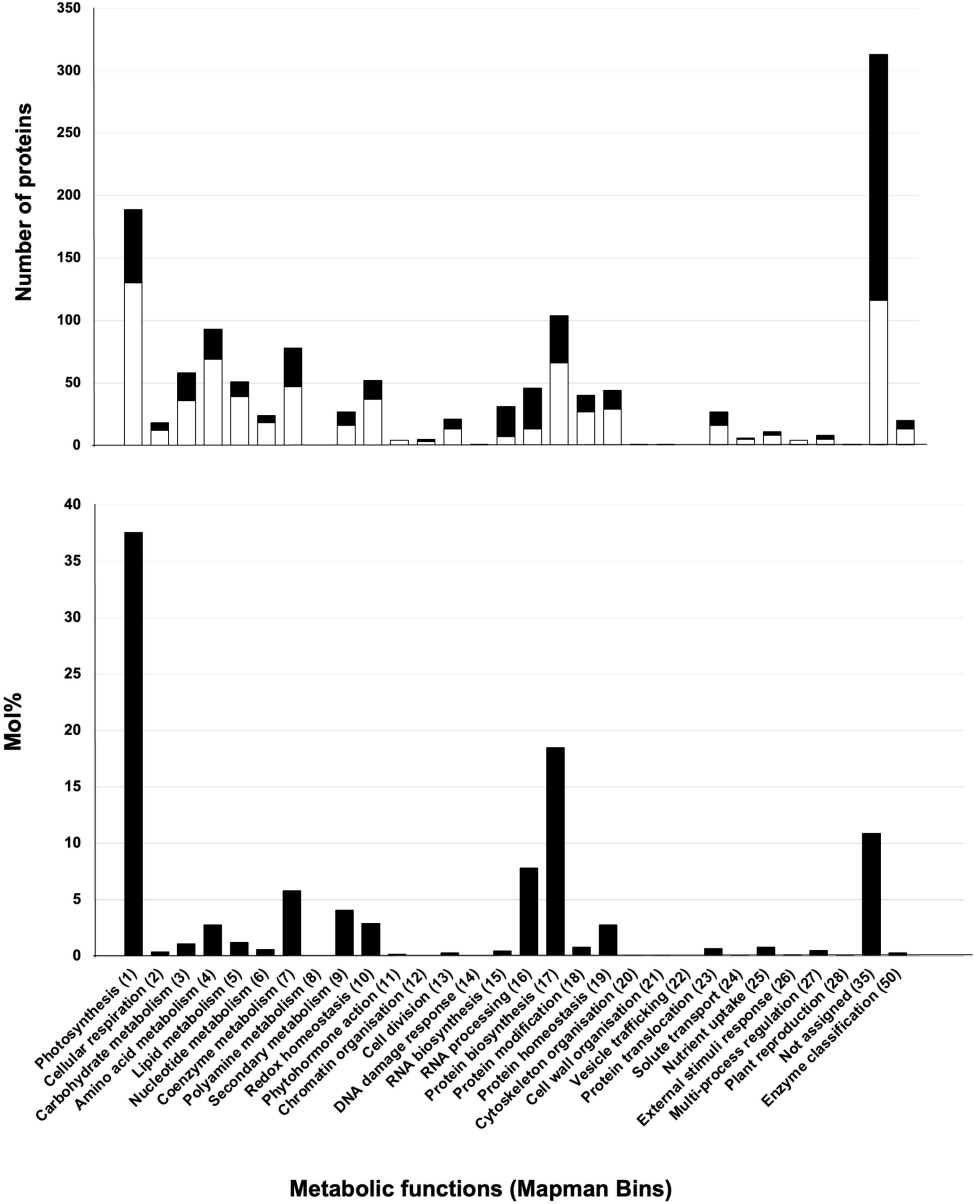
Functions and abundance of proteins detected in the tomato stromal proteome. The 1,278 proteins of the tomato stromal proteome were categorized into MapMan functional categories. MapMan bin numbers are within parentheses. The 733 proteins shared with the fruit plastid proteomes (white) and 545 proteins detected only in the leaf stromal proteome (black) are displayed. Proteins were detected in all but two MapMan bins. There were no proteins assigned to polyamine metabolism (Bin 8) or the vesicular trafficking (Bin 22) bins. The abundance of the proteins in each MapMan bin is displayed as mol % of the stromal proteome.

To infer function, stromal proteins were assigned MapMan function bins using Mercator (Lohse et al., 2014). Four of the five largest bins (>59 proteins) were associated with well-known chloroplast functions - photosystems, protein synthesis, amino acid metabolism, and RNA (**Figure 5** (top panel); **Table S5**). There was a surprising lack of correlation of numbers of proteins and the relative protein mass (based on mol %) for the top five bins (**Figure 5**) (bottom panel). For example, approximately 37.6% of the stromal protein mass was associated with the 94 proteins in the photosystems bin. In contrast, the 77 proteins in the RNA and the 94 proteins in the amino acid metabolism bins were 8.3% and 2.75% of the proteome, respectively. Manual curation of the proteins in the not-assigned bin (311 proteins) allowed specific or general functions to be assigned most proteins, leaving only 39 proteins as uncharacterized/unknown and 52 enzymes with unknown functions (**Tables S4D–F**). This curation grouped the stromal proteins into ten functional categories (**Table 4**; **Tables S5–S10**). Below we highlight several of these functional groups.

**Table 4:** Functional categories of proteins after manual annotation of the tomato leaf stromal proteome^A^.

Categories (# proteins)	Protein functions	Number of proteins	Table
**Chloroplast-genome encoded** **(44)**	Chloroplast genome encoded	44	**Table S5A**
**Photosynthesis** **(136)**	PSII	60	**Tables S5B-F**
	Cytb6/f	12	
	PSI	25	
	Ndh	28	
	ATPase	11	
**Plastid organization & division** **(50)**	Plastid division & Plastid differentiation	13	**Table S6A**
	Thylakoid biogenesis	13	
	Plastoglobules	13	
	Other Fibrillins	6	
	Miscellaneous	8	
**Photosynthetic Metabolism** **(164)^B^**	TCA cycle	21	**Tables S7A-I**
	Calvin cycle	40	
	OPP shunt	10	
	Major CHO	37	
	Minor CHO	17	
	C1 metabolism	7	
	Glycolysis & gluconeogenesis	23	
	Photorespiration	10	
	Paralogs	–	
**Non-Photosynthetic Metabolism** **(305)**	Amino acid biosynthesis genes	101	**Tables S8A-F**
	Nitrogen & Sulfur metabolism	28	
	Nucleotide metabolism	46	
	Cofactors & Vitamins	29	
	Lipid metabolism	57	
	Stress	52	
**Isoprenoid Metabolism** **(79)**	MEP pathway	10	**Tables S9A-C**
	*cis*-Prenyl transferases & terpene synthases	9	
	Carotenoids	15	
	Tocopherols & Plastoquinones	7	
	Tetrapyrroles	38	
**Redox homeostasis (51)**	Thioredoxins & Trx domain proteins	22	**Table S9D**
	Peoxiredoxins	4	
	Glutathione peroxidases	7	
	Ferridoxin-thioredoxin proteins	2	
	Superoxide dismutase	5	
	Ascorbate & glutathione	11	
**Protein synthesis & homeostasis (316)**	Translation	140	**Tables S10A-E**
	Protein import	32	
	Protein folding	61	
	Post-translational modifications	36	
	Proteolysis	53	
**Transcriptional & post-transcriptional regulation** **(168)**	Nucleoid	58	**Tables S6B-F**
	Transcription factors & regulators (not nucleoid associated)	10	
	Other DNA-binding proteins	9	
	RNA-processing & RNA-binding proteins	84	
	Signaling proteins	9	
**Uncharacterized or unknown function** **(99)**	Uncharacterized proteins	39	**Tables S4D-F**
	Miscellaneous enzymes of unknown function	52	
	Miscellaneous enzymes	11	
**Miscellaneous**	Membrane proteins	159	**Tables S4A-C**
	Lumenal proteins	59	
	Arabidopsis top 23 stromal proteins & tomato orthologs	–	

^A^ All proteins in the stromal proteome were manually annotated (see Methods). Based on published functions for tomato proteins or their orthologs in *Arabidopsis thaliana*, proteins were organized in functional categories. Some proteins appear in more than one category.

^B^ A total of 161 proteins are listed in the C metabolism workbook; however, two proteins (SHM1 & SGPP) appear in two worksheets due to their roles in multiple processes.

### Photosynthetic complexes

Over 19.8% of the stromal proteome mass was associated with the major multimeric photosynthetic complexes - photosystem I-Light Harvesting Complex I (PSI-LHCI), PSII-LHCII, cytochrome b6f, ATP synthase, and NADH dehydrogenase (NDH) complexes – and the proteins involved in complex stability and assembly (**Table S5**). Photosynthesis initiates with the absorbance of light energy by light-harvesting complex proteins (LHCII) and photosystem II (PSII) (Buchanan et al., 2015). The vast majority of PSII-associated proteins are integral-membrane proteins and were not detected (**Table S5B**). The chloroplast genome-encoded PSBA-E were detected infrequently, at low levels and with non-molar ratios. The nuclear-genome encoded PSII subunits (PSBR and PSBS) and five LHCII subunits (LHCB13, 1A, 1B, 3C, and CP29.1) were detected at low levels (≤0.007 and ≤0.006 mol%, respectively) and sporadically identified. Whereas PSB33 and LHCB9 were 2.6- and 3.2-fold more abundant, respectively, and detected in all samples analyzed, suggesting a looser association with the thylakoid membranes. The most abundant proteins were the lumenal oxygen-evolving proteins (PSBO-1, PSBO-2, PSBP, and PSBQ). Dozens of proteins important for PSII protein and pigment assembly, stability or repair are known in Arabidopsis (Lu, 2016; Liu and Last, 2017; Sato et al., 2017; de Luna-Valdez et al., 2019; Li et al., 2019). We detected 29 of these orthologous proteins, as well as thio/disulfide-modulating proteins critical for PSII assembly/maintenance and protein processing/turnover (**Table S5B**; **Table 4**).

Linking PSI and PSII, the cytochrome b6/f complex has eight subunits (Malone et al., 2019) and two integral membrane proteins (PETA and PETB) and the lumenal PETC were detected. All proteins associated with photosynthetic electron transport (PETE, PETJ, two PETFs, and two PETHs) were detected (**Table S5C**). PETH1 is the most abundant of these proteins (0.27 mol%) (**Table 2**) and is 6-fold more abundant than its paralog. Two complex assembly/stability factors were detected - HCF164 and LIR1 (Yang et al., 2016; Lennartz et al., 2001) (**Table S5C**).

PSI and its light-harvesting complex is an asymmetric assemblage of 15 PSI proteins, LHCA proteins, and PSI assembly proteins (**Table S5D**) (Amunts et al., 2010). We detected 11 PSI subunits including six integral membrane proteins (PSA-A, B, F, G, K, and L) and extrinsic proteins exposed on the stromal (PSA-C, D, E) and lumenal (PSA-N) side. Of the PSI-associated LHC proteins, proteins similar to AtLHCA1 and AtLHCA2 were not detected, but two AtLHCA3-like (LHCA8A, LHCA8B) and one AtLHCA4-like (LHCA11) proteins were detected. Finally, we detected five PSI assembly proteins including: YCF3, YCF3-interacting factor, PPD1, PSA2, and PSA3 (Naver et al., 2001; Liu et al., 2012; Fristedt et al., 2014; Shen et al., 2017; Nellaepalli et al., 2018). Notably, the chloroplast genome-encoded YCF4 was not detected, although it was detected in the tomato fruit proteome (Barsan et al., 2012) (**Table S3**).

The NAD(P)H-dehydrogenase-like complex (NDH) associates with two PSI complexes and is active in photorespiration (Shikanai, 2016), as well cyclic electron flow to preferentially contribute to ATP synthesis (Munekage et al., 2002; Yamamoto and Shikanai, 2019). Proteins associated with electron flow (PGR5, PGRL1A and PIFI) were detected, as were many subunits of the NDH complex and several NDH assembly proteins (**Table S5E**) (Wang and Portis, 2007; Shikanai, 2016). NDH is the largest complex with 29 proteins organized into subcomplexes (Shikanai, 2016). Six NDH subunits of the stroma-facing of subcomplex A (NDH-H, I, J, M, N, O) and five proteins critical for assembly (CRR1, 6, 7, 9, and 41) were detected (**Table S5E**). In addition, subcomplex E (NDH-S, U, and V) and all subunits of stroma-exposed subcomplex B (PNSB1-PNSB5) and lumenal subcomplex L (PNSL1-PNSL5) were detected; whereas, none of the proteins in the thylakoid membrane-associated subcomplexes SubL nor SubM were detected. The subunits for the NDH subcomplexes A, B and L were not detected in equimolar ratios. Finally, the minor LHCA proteins (similar to AtLHCA5 and AtLHCA6) that mediate the PSI-NDH super-complex formation were not detected (Peng et al., 2009).

The ATP synthase complex is composed of eight different subunits (Hahn et al., 2018). All subunits of the extrinsic CF1 complex, which are peripheral thylakoid membrane proteins, were detected (**Table S5F**). While ATPA, ATPB, ATPD, ATPE, and ATPC are present in a 3:3:1:1:1 ratio in CF1, their abundance in the tomato stroma did not reflect this stoichiometry. ATPB was 27-, 32-, 22-, and 55-fold more abundant than ATPA, ATPD, ATPE, and ATPC, respectively. While the integral membrane subunits ATPH and ATPI were not detected, the ATPF and ATPG subunits, which are tethered to ATPH, were detected at substantially lower levels than the CF1 complex proteins (**Table S5F**). Finally, four ATP synthase biogenesis proteins were detected (ALB4, BFA1, BFA2, and PAB) (Mao et al., 2015; Trösch et al., 2015; Zhang et al., 2016; Zhang et al., 2018). In contrast, the assembly proteins P11 (Solyc02g093690) and P12 (Solyc02g031770) were not detected (Duan et al., 2020).

Of critical importance to the function of the photosynthetic complexes is the biogenesis and maintenance of the thylakoid membranes. In addition, proteins associated with plastid fission, chloroplast differentiation, and plastoglobules are important for chloroplast structure and function (**Table S6**). Of the 53 proteins in this group, 19 were fibrillins (Laizet et al., 2004). Ten different types of fibrillins were detected in 87-100% of the samples and ranging from 0.06 mol % (FBN-like) to 0.07 mol % (FBN4).

### Photosynthetic metabolism in chloroplasts

The chloroplast is a metabolic hub synthesizing a broad spectrum of molecules essential for plant growth, development and adaptation to stress (Rolland et al., 2012; Buchanan et al., 2015). A significant proportion of the tomato stromal proteome was associated with the central (or primary) metabolic pathways of photosynthetic metabolism (Wise and Hooper, 2006). These pathways include the Calvin cycle, TCA cycle, OPP pathway, major and minor carbohydrate metabolism, C1 metabolism, glycolysis, gluconeogenesis, and photorespiration (**Table 4**, **Tables S7A–H**). A total of 165 proteins associated with carbon metabolism were detected and, collectively, they constituted 19.3 mol % of the stromal proteome. Notably, 27 of these proteins were encoded by single-copy genes in Arabidopsis and by two paralogs in tomato (**Table S7I**). The majority of the paralogous proteins accumulated to different levels in the tomato stroma ranging from 1.1- to 161-fold different. For example, the RuBisCo large subunit methyl transferase LMST2 was 48-fold more abundant than LMST1 (**Table S7I**). These data suggest that the duplicated genes have allowed for changes in paralog abundance and, potentially, in function.

### Non-photosynthetic metabolism in plastids: amino acids, nitrogen, sulfur, nucleotides, co-factors, and vitamins

Numerous non-photosynthetic central metabolic pathways are active within chloroplasts including N and S metabolism and biosynthesis of nucleotides, co-factors and vitamins, amino acids, lipids, and defense-associated oxylipins (**Table S8A**). We also detected 11 enzymes with roles in other metabolic pathways and identified 52 enzymes that could not be reliably assigned to a pathway (**Tables S4E, F**).

The largest group of proteins associated with non-photosynthetic central metabolism were the 101 enzymes that catalyze amino acid biosynthesis (Lancien et al., 2007) (**Table S8A**). Four enzymes associated with aromatic amino acid (TSA, SK, ASB2) or histidine (HIS-N5B) biosynthesis were identified in the stroma for the first time (**Table 3**). In addition, three ACT-domain proteins with unknown function were identified; ACT domains bind amino acids and are often used in amino acid feedback-regulated enzymes.

The chloroplast contributes to N and S metabolism (**Table S8B**) (Masclaux-Daubresse et al., 2010; Chan et al., 2013). Seven enzymes in N metabolism were detected with glutamine synthase 2 (GS2) being most abundant (0.4 mol %). We detected 21 proteins associated with S metabolism, which centers on Cys biosynthesis and catabolism. Cys is essential for protein biosynthesis and is a critical residue in enzyme active sites, protein tertiary structure, protein-protein interactions, redox sensitive enzyme activity, [Fe-S] groups, vitamins, and cofactors (**Table S8B**). Proteins involved in sulfate catabolism (APS1, APR3, SiR), Cys biosynthesis (OASC, CS26), as well as Cys-derived methionine (GS, CBL), cystathione (CBX1A-C), and glutathione (GSH1, GSH2) biosynthesis were detected. Finally, SAL1, a critical redox-responsive regulator of the retrograde stress signal PAP, was detected (Chan et al., 2013); while the integral-membrane antiporters of PAPS/PAP (PAPST1 and PAPST2) that help to control the levels of cytosolic PAP were not detected (Ashykhmina et al., 2019).

Forty-six enzymes associated with nucleotide metabolism were detected (**Table S8C**). Of these, six were detected for the first time, including an Appr-1-p processing domain protein (Kumaran, 2005), a nucleoside diphosphate kinase (NDK3) and a ribose-phosphate pyrophosphokinase 3 (PRS3) (**Table 3**). Surprisingly, we reproducibly detected two enzymes of pyrimidine biosynthesis, dihydroorotate dehydrogenase (DHODH) and orotidine 5’-phosphate decarboxylase (ODCase), which catalyze tandem steps in pyrimidine biosynthesis in the stroma. The tomato DHODH had no predicted targeting signals and was previously detected in plant mitochondria (Bellin et al., 2021). In contrast, the tomato ODCase had strong predictors for plastid localization (**Table S8C**); although previous studies suggest it resides in the cytosol. The stromal localization of both proteins may provide new insights into pyrimidine metabolism in tomato.

### Non-photosynthetic metabolism: lipids and oxylipins

The central metabolic pathways for lipids and phytohormone biosynthesis are highly conserved (Li-Beisson et al., 2013; Wasternack and Song, 2017). Fifty-seven enzymes associated with lipid metabolism (1.24 mol %) were identified (**Table S8E**). Enzymes for the synthesis of acetyl-CoA (ACS and the pyruvate dehydrogenase complex), all soluble enzymes for lipid elongation, many lipases, and lipid-binding proteins were detected. The inner membrane-associated enzymes and enzymes associated with lipid desaturation were not detected. An acyl carrier protein (ACP5) and the oleoyl-acyl carrier protein thioesterase 2 (FATA) were not previously reported in the Arabidopsis proteomics databases (**Table 3**; **Table S8E**). The enzymes essential for the synthesis of jasmonic acid (JA), which is critical for plant defense and development, and numerous oxylipins with roles in defense signaling including the HPL branch that produces C6 volatiles were detected (**Table S8E**) including two lipoxygenases (LOXC and LOXF), allene oxide synthase (AOS), allene oxide cyclase (AOC), and hydroperoxide lyase (HPL).

### Isoprenoid metabolism, retrograde signals, and other metabolic pathways

Isoprenoids are the largest and most diverse group of natural products in plants, with over 35,000 different compounds (Kirby and Keasling, 2009). The plastid-derived isoprenoid metabolites (heme, chlorophylls, carotenoids, ABA, gibberellins, strigolactones, plastoquinones, phylloquinones, tocopherols, and terpenoid volatiles) are derived from the five-carbon isopentenyl diphosphate (IPP) and DMAPP, which are primarily synthesized by the MEP pathway (Zhou and Pichersky, 2020). Seventy-nine proteins associated with isoprenoid production were detected in the stromal proteome (**Tables S9A-C**). All enzymes of the plastidial MEP pathway, as well as two IPP isomerases, were detected. DXS, which creates the substrates for the MEP pathway and thiamine biosynthesis, is encoded by two tomato paralogs. DXS1 was 17-fold more abundant than DXS2 in leaf chloroplasts (**Table S9A**), which consistent with *DXS1* and *DXS2* RNA levels in leaves and fruit (Paetzold et al., 2010). Additional enzymes detected included three *cis*-prenyl transferases, two geranylgeranyl pyrophosphate synthases (GGPPS), a GGPPS small subunit (SSU-II), and three terpene synthases (**Table S9A**). While Barja et al. (2021) and Zhou and Pichersky (2020) reported three plastidial GGPP synthases (SIG1-3) with similar kinetic parameters, only SlG2 and SlG3 were detected in our leaf stromal proteome. The absence of SIG1 protein (Solyc11g011240) was consistent with low levels of *SIG1* mRNAs, relative to *SIG2* and *SIG3* (Barja et al., 2021). It is also noteworthy that SSU-I (Solyc07g064660), which is known to modify SIG1-3 activity was not detected (Zhou and Pichersky, 2020).

GGPP is used for the synthesis of carotenoids, which are important for stabilization of the photosynthetic apparatus, light capture, and photoprotection (Stanley and Yuan, 2019). The carotenoid-derived apocarotenoids are important for synthesis of abscisic acid and strigolactone, as well as producing a suite of volatiles important in development and stress signaling (e.g., β-cyclocitral). Fifteen enzymes associated with carotenoid metabolism were detected; although the rating-limiting leaf phytoene synthase 1 (PSY1), orange chaperones, and carotenoid-cleavage enzymes were not detected (**Table S9B**).

GGPP is also used to synthesize tocopherols, chlorophylls, plastoquinones, and phylloquinones (**Table S9B**). Tocopherols scavenge singlet oxygen (^1^O_2_) derived from photosynthesis. The biosynthetic enzymes (VTE1, VTE3, and VTE4) and regulatory kinases (ABCK1 and ABCK3) were detected (**Tables S9B**). In addition, the plastoquinione biosynthesis enzyme, solanesyl diphosphate synthase, was detected. The tetrapyrrole pathway yields hemes, the chlorophylls for the PSI and PSII light-harvesting antennae, and protochlorophyllide (PChlide), which is a critical photosensor role in chloroplast-nuclear communication. We detected 38 enzymes associated with tetrapyrrole biosynthesis and catabolism (**Table S9C**). The complete tetrapyrrole pathway was represented with the exception of the membrane-bound chlorophyllide A oxygenase and uroporphyrinogen III methylase. Tomato also has expanded its tetrapyrrole protein complement with two *UROD* and three *POR* paralogs (**Table S9C**) (Gabruk and Mysliwa-Kurdziel, 2020).

PChlide is a photosensitizer that is critical in retrograde signaling (de Souza et al., 2017). By transferring its excitation energy to oxygen, PChlide creates the highly reactive ^1^O_2_. To limit ^1^O_2_ production and photosensitivity, AtFLU controls PChlide levels (op den Camp et al., 2003). We detected two FLU proteins (FLU1 and FLU2) that are 64% identical and FLU1 is 4-fold more abundant than FLU2. Neither have been studied to date and it is unclear if they are functionally redundant (**Table S9C**). In Arabidopsis, the EXECUTER proteins (AtEX1 and AtEX2) have critical but distinct roles in perception of ^1^O_2_ and triggering the reprogramming of nuclear gene expression for stress adaptation (Lee et al., 2007; Dogra et al., 2017; Duan et al., 2019). In tomato, EX2 is 8-fold more abundant than EX1 (**Table S9C**), which may reflect differences in the roles of the tomato EX proteins, the tightness of association or location within the grana margins of the thylakoid. Finally, SAFEGUARD1, which suppress ^1^O_2_ production at the thylakoid grana margins (Wang L. et al., 2020) is 1.8-fold more abundant than EX2 (**Table S9C**).

### Redox regulation: damage control to cellular homeostasis

Chloroplasts use redox-regulatory systems to limit cellular damage from ROS and adapt plant metabolism to fluctuating light/dark cycles and environmental insults, such as abiotic stress or pathogen/pest attack (Exposito-Rodriguez et al., 2017; Cejudo et al., 2019; Yoshida et al., 2019; Fichman and Mittler, 2020). Redox regulation is dependent on the electron transport chain of the thylakoid’s photosynthetic complexes to produce reducing power, which is transferred from ferredoxin (Fd) to a thioredoxin (Trx) via Fd-Trx reductase (FTR). The diversity of proteins with Trx and Trx-like motifs and down-stream redox proteins provides flexibility and specificity in responses. We identified 51 redox-regulation proteins including: FTRs, thioredoxin domain-proteins, peroxiredoxins, glutathione peroxidases, superoxide dismutases, ascorbate/glutathione cycle proteins, and proteins with a cystathionine β-synthase (CBX) domain (**Table S9D**). The abundance of the redox proteins varied within a 1020-fold range with Fe-SOD2A (1.01 mol %) as the most abundant protein. The tomato redox systems are distinguished from Arabidopsis by the facts that: (1) the tomato Trx-m4 family is expanded (three paralogs), (2) there are two NTRC proteins (with one detected), (3) there are two Fe-SOD2 paralogs, (4) the 2-CYS-Prxs collectively are the most abundant peroxiredoxin in the tomato stroma, but their abundance is significantly lower than in Arabidopsis (**Table S4C**), and (5) the CBX1 protein family (with probable roles regulation of redox signaling) is expanded (three paralogs) (**Table S9D**, **Table 3**) (Cheng et al., 2014).

### Protein homeostasis

Approximately 3,000 plastid-localized proteins are encoded by nuclear genes, translated on cytosolic ribosomes and imported into plastids (Thomson et al., 2020), while the remaining 81 proteins are synthesized on chloroplast ribosomes (Daniell et al., 2006; Kahlau et al., 2006). Within the chloroplast, proteins must be folded, post-translationally modified, transported to their sub-compartment within the chloroplast, associated with their cofactors, assembled into their multimeric complexes, and ultimately be targeted for proteolytic turnover. Protein homeostasis is carefully regulated to ensure metabolic responses are coordinated with light/dark cycles and can adapt to the stresses imposed by PS-generated ROS and the environment. Not surprisingly, we detected over 322 proteins that orchestrate the life and death of proteins (**Table S10**).

The plastid’s 50S and 30S ribosome complexes are essential for synthesizing chloroplast genome-encoded proteins. Perturbations in translation are perceived and communicated to the nucleus (via GUN1) to coordinate plastid biogenesis and mediate adaptation to stress (Marino et al., 2019; Wu et al., 2019). We detected 33 RPL subunits, 23 RPS subunits, 5 plastid-specific ribosomal proteins (PRSPs), as well as 29 proteins were associated with rRNA, tRNA, or ribosomal protein modifications (**Table S10A**). The ribosomal protein subunits were not present at equimolar levels. Six subunits were particularly abundant including the chloroplast genome-encoded RPS19, RPS15 and RPL23 and nuclear-genome encoded RPL12A, RPL12B and RPS1A. In addition, 27 amino-acyl tRNA synthases and 20 proteins associated with translational initiation, elongation, termination or regulation were identified. Seven of the tRNA synthases lacked an identifiable transit peptide, while 17 had predicted chloroplast or mitochondrial transit peptides (**Table S10A**). If similar to Arabidopsis, many of these proteins may have dual localization in the chloroplast and mitochondrion or cytosol (Duchene et al., 2005).

Import of proteins into plastids is a regulated process and disruption of import provides a retrograde signal to mediate stress adaptation (Wu et al., 2019). There are several routes for entry into the chloroplast including the canonical import via the outer and inner membranes (TOC and TIC complexes) and inter-organellar channels (Cline and Dabney-Smith, 2008; Armbruster et al., 2009; Nakai, 2018; Thomson et al., 2020). We identified 32 proteins involved in subcellular targeting (**Table S10B**). Few of the membrane-associated TOC/TIC translocation machinery proteins were detected, while the associated chaperones were readily detected. The proteases (PREP1, SPP, TOP1) that remove the N-terminal transit peptide from imported proteins (**Table S10E**) and ten other proteins critical for translocating proteins into the thylakoid membrane or lumen were also identified (**Table S10B**).

To establish and maintain their secondary, tertiary, and quaternary structures to preserve protein function, the chloroplast has an impressive array proteins to facilitate protein folding with 61 different proteins identified in the tomato stroma (**Table S10C**). This included: 30 chaperones or chaperonins; three ATP-dependent chaperones of the Clp protease (ClpC1, ClpC2, and ClpD), the ClpB3 disaggregase, 19 peptidyl-prolyl *cis-trans* isomerases, and seven protein disulfide isomerases. Three of these proteins (DJC65, DJC73 and FKBP17-1) were not previously detected (**Table 3**).

Proteins are also post-translationally modified by addition/removal of chemical moieties or by proteolytic processing to influence protein function or stability. We detected 36 modification enzymes in six functional categories: kinases, phosphatases, methylases, acetylases, deformylases (PDFs), and peptide methionine sulfoxide reductases (**Table S10D**) and 13 N-terminal peptidases (**Tables S10D, E**) (Walling, 2006; Gibbs et al., 2016). Unique to tomato are the wound-induced LAPs (LAP-A1 and LAP-A2) that control the expression of nuclear genes associated with the wound- and stress-responses via their aminopeptidase and/or chaperone activities (**Table 3**) (Gu and Walling, 2000; Fowler et al., 2009; Scranton et al., 2012; Scranton et al., 2013).

The chloroplast also has a robust complement of oligopeptidases and endoproteases to mediate protein turnover (Kmiec et al., 2014; Nishimura et al., 2017). These proteinases and proteolytic complexes are located within envelope, stroma, lumen, or thylakoid membranes. We detected a total of 53 proteins associated with proteolysis (2.6 mol %) (**Table S10E**). While these proteins primarily remove damaged or unfolded proteins from the chloroplast, it is also clear that peptidase activity is critical for chloroplast signaling, as evidenced by the requirement of FtsH2 protease-mediated turnover of EX1 for signaling ^1^O_2_ damage (Wang et al., 2016), role of LapA in tomato defense gene expression (Fowler et al., 2009), and role of chloroplast peptides in defense signaling (Kmiec et al., 2018).

The stroma-localized Clp complex is well characterized structurally and known to have a critical role in protein homeostasis and proteome remodeling (Nishimura et al., 2017; Rowland et al., 2022). We detected all subunits of the stromal Clp complex (**Table S10E**), three Clp chaperones, as well as the ClpS, ClpF, ClpT1, and ClpT2 proteins that help deliver or provide substrate specificity to the Clp protease (Nishimura et al., 2017). The tomato has two ClpC paralogs with ClpC1 being 2.7-fold more abundant than ClpC2.

Little is known of the function of tomato’s chloroplast DEG proteases (**Table S10E**) (Nishimura et al., 2017). We detected two stromal DEG2 paralogs in tomato, and three lumenal DEGs (DEG1, DEG5 and DEG8), but the stromal DEG7 (Solyc02g091410) was not detected. The filamentation temperature-sensitive H (FtsH) proteases are associated with membranes, turnover of proteins damaged by ROS, and thermotolerance. In tomato, the thylakoid FtsH6 has a role in thermotolerance (Sun et al., 2006). Of the nine FtsH proteins, the thylakoid-localized (FtsH2 and FtsH5) and inner envelope-localized (FtsH7 and FtsH11) were detected (**Table S10E**). If similar to the AtFtsH2, the tomato FtsH2 may be critical for retrograde signaling by controlling the turnover of D1 (a reaction center protein of PSII) and the ^1^O_2_ sensor EX1 at the margins of the grana (Wang et al., 2016). Finally, three C-terminal processing peptidases (CTPA1-3) and two subunits of the EGY (ethylene-dependent gravitropism-deficient and yellow-green) protease were detected.

### The replication and transcriptional hub of the chloroplast

The proteomes of nucleoids and transcriptionally active chromosomes (pTAC) from plastids are influenced by the differentiation state of plastids and/or environmental factors and have been characterized in Arabidopsis and maize (Huang M. S. et al., 2013; Melonek et al., 2016). We detected 58 nucleoid- and TAC-associated proteins (**Table S6B**). This included all plastid-encoded RNA polymerase (PEP) subunits, 20 PEP-associated proteins, nine DNA replication and repair proteins, four redox proteins, ten RNA biogenesis enzymes, two kinases, and six other proteins with diverse functions. Surprisingly, we did not detect the seven sigma factors (SigA-F) that interact with PEP. Collectively, the nucleoid/pTAC proteins detected in the tomato stroma constituted 2.32 mol % of the proteome ranging from 1.02 mol % (Fe-SOD2A) to 0.0002 mol % (DNA topoisomerase) (**Table S6B**).

For the conserved nucleoid core, we detected the MURE-like protein and all but three (pTAC9, pTAC11 and pTAC13) of the 18 pTACs (Melonek et al., 2016)(**Table S6B**). While tomato genome has pTAC9 (OSB2, Solyc09g007430) and pTAC13 (Solyc09g011830) genes, it does not encode a pTAC11-like protein (WHIRLY3) (Akbudak and Filiz, 2019). We detected pTAC7, pTAC10, pTAC12, and pTAC14, as well as the FNL1 and FNL2 kinases, that are known to interact with one another to regulate the activity of PEP (Gao et al., 2012; Huang C. et al., 2013; Chang et al., 2017). While the function of pTAC17 is unknown, we detected two tomato pTAC17s; the tomato pTAC17A was the most abundant pTAC protein identified (0.07 mol %) and was 106-fold more abundant than pTAC17B.

In addition to the proteins associated with transcriptionally active nucleoids, we detected proteins involved with DNA replication, chromatin assembly, recombination, transcription factors, RNA processing and binding, and signaling (**Tables S6C-F**). There were 82 proteins important for post-transcriptional control (**Table S6E**). While there is substantial evidence for transcription factors being dual-localized in Arabidopsis, only ten transcription factors and regulators were detected (**Tables S6B, C**) (Krause et al., 2012; Krupinska et al., 2020). Three histone proteins (two H3-2 proteins and one H2B.1) were detected; their roles within the chloroplast are unknown (**Table S6D**).

## Discussion

The tomato stromal proteome is an important contribution to the field of plastid proteomics, providing novel insights into the protein complement of a eudicot’s stroma, as few stromal proteomes are currently available (Peltier et al., 2006; Olinares et al., 2010). The unprecedented depth of the tomato stromal proteome with 1,278 rigorously identified proteins was achieved due to the purity of our stromal preparations (Bhattacharya et al., 2020) and accuracy and sensitivity of the Orbitrap Fusion MS. Our data complements the plastid proteomes of tomato fruit and leaves (Barsan et al., 2010; Barsan et al., 2012; Tamburino et al., 2017), as well as Arabidopsis stromal proteomes (Peltier et al., 2006; Olinares et al., 2010). Our endeavors provided empirical evidence for 545 tomato plastid proteins and 92 Arabidopsis stromal proteins that were not previously reported (Sun et al., 2009; Barsan et al., 2012). Furthermore, using emPAI as a measure of protein abundance, we showed that when the most abundant proteins in the tomato vs Arabidopsis stroma were compared, there were significant differences in the abundance of orthologous proteins suggesting that the mechanisms that regulate protein homeostasis may have diverged in these model plants. This diversity has the potential to impact the ability of a plastid to sense and transmit signals to inform organellar networks of deviations from plastidial and cellular homeostasis (de Souza et al., 2017; Fernandez and Burch-Smith, 2019; Unal et al., 2020; Wang Y. et al., 2020).

One of these diverged protein homeostasis mechanisms is likely to involve the Solanaceae-specific, wound-induced and stromal LAP-A (Chao et al., 1999; Narváez-Vásquez et al., 2008; Fowler et al., 2009; Scranton et al., 2012) (**Table 3**). LAP-A upregulates nuclear-genome encoded genes associated with the late branch of wound signaling; LAP-A acts downstream of JA perception and accumulation (Fowler et al., 2009) and, also, downregulates a set of stress-response genes (Scranton et al., 2013). Given LAP-A’s residence in the stroma and ability to modulate nuclear gene expression, LAP-A appears to generate a signal to enable chloroplast-nucleus communication and, thereby, deploy adaptations to cope with ROS, mechanical damage, herbivory, and pathogen attack. To understand its global impact on tomato defense and chloroplast-to-nucleus signaling, the tomato stromal proteome sets the foundations for the multi-omics approaches that are being pursued to characterize of the MeJA- and LAP-A-dependent proteome, N-terminome, metabolome, and transcriptome.

Well studied in Arabidopsis, less is known about retrograde signaling in crops (de Souza et al., 2017; Marino et al., 2019). The tomato stromal proteome provided empirical evidence for accumulation of proteins associated with the synthesis of plastidial metabolites known as retrograde signals including proteins associated with sulfur (PAP, 3’-phosphoadenosine 5’-phosphate), carotenoid (β-cyclocitral), isoprenoid (MEcPP, 2-C-methyl-D-erythritol 2, 4-cyclodiphosphate), and fatty acid metabolism (**Table S9**). In addition, a robust complement of proteins associated the generation and dissipation of reactive oxygen species (ROS) or serving as photosensitizers (tetrapyrroles, FLU, EX), as well as protein homeostasis were identified (**Tables S8, S9**). The manual curation of the proteins of tomato stroma identified additional diversity that may be important for the ability of tomato chloroplasts to act as stress sensors and modulate these operational retrograde signals allowing rapid adaptation to biotic and abiotic stress. Significantly, tomato had expansions of some of these gene families and there were substantial differences in protein abundance between paralogs. Examples, included 27 proteins associated with: photosynthetic metabolism, redox and ROS scavenging (NTRC1/NTRC2 and Trx-domain proteins), tetrapyrrole accumulation (UROD1/2, POR1/2/3, FLU1/FLU2) and perception of ^1^O_2_ (EX1/EX2), protein homeostasis (ClpC1/C2, LAP-A1/A2, DEG2A/2B, CPN20) and regulation of transcriptionally active chromosomes (pTAC17A/B) (**Tables S7, S9, S10**). These discoveries present new avenues for understanding the biochemical and signaling complexities of tomato’s stromal compartment.

## Data availability statement

The datasets presented in this study can be found in online repositories. The names of the repository/repositories and accession number(s) can be found below: www.proteomexchange.org, PXD035944.

## Author contributions

OB isolated tomato chloroplasts and purified stromal proteins. NH enabled critical analysis of datasets. IO created the tomato chloroplast protein Atlas and assembled the data from Arabidopsis databases, NCBI and Mercator. IO, OB and LW performed data analysis and manual curation. OB, IO and LW wrote the manuscript collaboratively. All authors read and approved the final version of the manuscript.

## Supplementary material

Supplementary Figure 1 SDS-PAGE gels, gel fragment excision and LC-MS/MS analysis. Stromal proteins were isolated as described by Bhattacharya et al. (2020) and fractionated by 12% SDS PAGE. Gels were stained with Coomassie blue. The MW markers and stromal proteins were run on the same gel and two intervening lanes were excised for this figure. The gel section with 50- to 75-kDa proteins was excised (see the arrows). The <50-kDa sections was separated into two fractions containing proteins that were between < 20-kDa (low mass) and a fraction that had proteins from <50-kD and > 20-kDa (intermediate mass). The low mass proteins were pooled with the > 70-kDa (high mass) proteins. The low/high and intermediate mass pools had nearly equivalent amounts of protein and were processed for LC-MS/MS as described in Methods.
