# Supplementary figures and images for: The tomato chloroplast stromal proteome compendium elucidated by leveraging a plastid protein-localization prediction Atlas
Source: Front Plant Sci. 2023 Aug 28;14:1020275. doi: 10.3389/fpls.2023.1020275 (PMC10493611; doi:10.3389/fpls.2023.1020275)

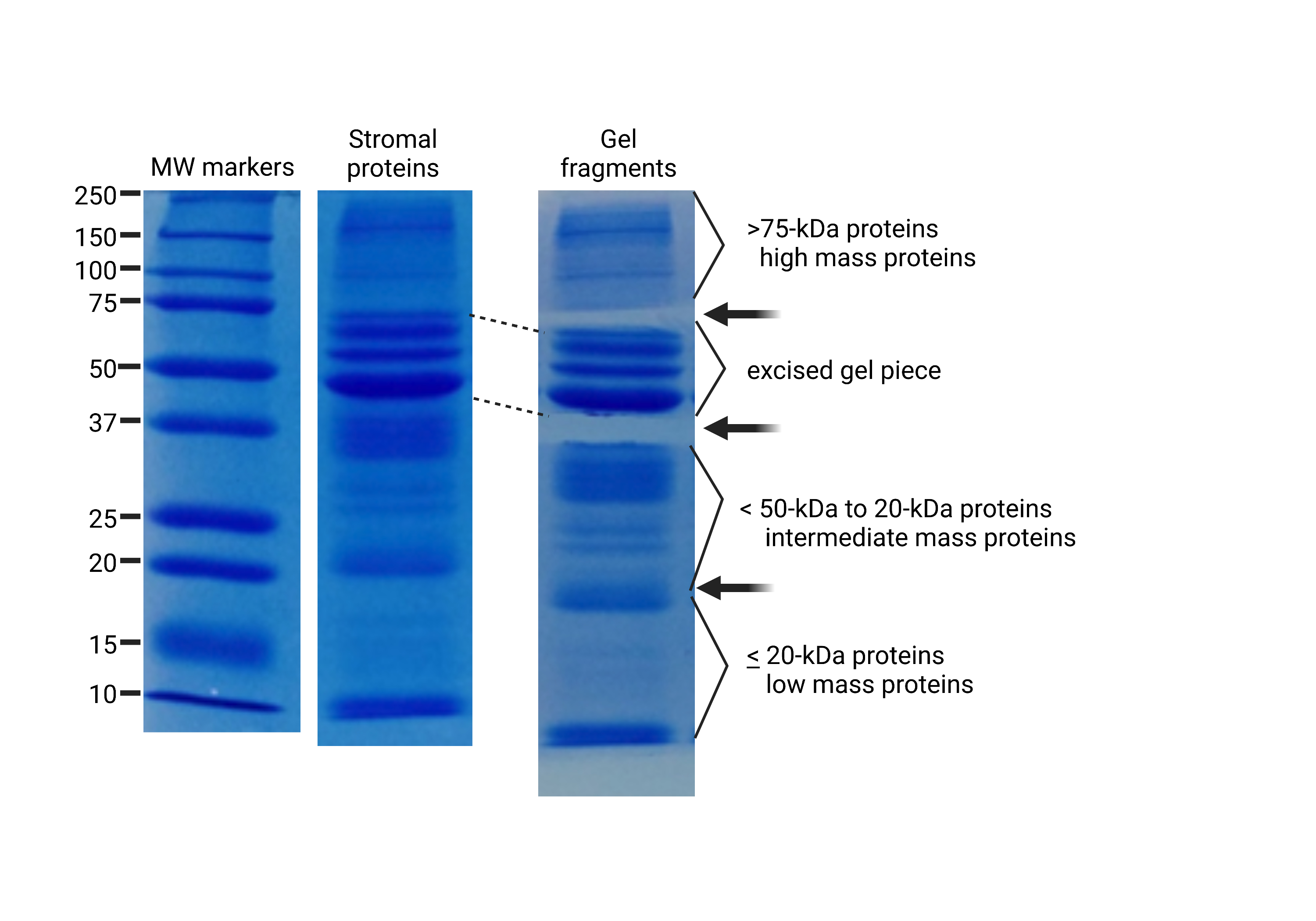

Supplement: Supplementary Figure 1 — SDS-PAGE gels, gel fragment excision and LC-MS/MS analysis. Stromal proteins were isolated as described by Bhattacharya et al. (2020) and fractionated by 12% SDS PAGE. Gels were stained with Coomassie blue. The MW markers and stromal proteins were run on the same gel and two intervening lanes were excised for this figure. The gel section with 50- to 75-kDa proteins was excised (see the arrows). The <50-kDa sections was separated into two fractions containing proteins that were between < 20-kDa (low mass) and a fraction that had proteins from <50-kD and > 20-kDa (intermediate mass). The low mass proteins were pooled with the > 70-kDa (high mass) proteins. The low/high and intermediate mass pools had nearly equivalent amounts of protein and were processed for LC-MS/MS as described in Methods. [file Image_1.jpg]
