# Supplementary material for: The tomato chloroplast stromal proteome compendium elucidated by leveraging a plastid protein-localization prediction Atlas
Source: Front Plant Sci. 2023 Aug 28;14:1020275. doi: 10.3389/fpls.2023.1020275 (PMC10493611; doi:10.3389/fpls.2023.1020275)
Supplement: Supplementary file 2 [file DataSheet_1.docx]

**Reference List for Supplementary Tables S2-S10**

Precedents for protein/gene names in the literature were searched for. These names and the papers that create the naming precedent are provided in supplementary Tables S2-S15 and are listed below.

Andolfo, G., Ruocco, M., Di Donato, A., Frusciante, L., Lorito, M., Scala, F. et al. (2015) Genetic variability and evolutionary diversification of membrane ABC transporters in plants. *BMC Plant Biol. 1*5, 51. doi:10.1186/s12870-014-0323-2

Barja, M.V., Ezquerro, M., Beretta, S., Diretto, G., Florez-Sarasa, I., Feixes, E. et al. (2021) Several geranylgeranyl diphosphate synthase isoforms supply metabolic substrates for carotenoid biosynthesis in tomato. *New Phytol.* 231, 255-272. doi:10.1111/nph.17283

Barsan, C., Zouine, M., Maza, E., Bian, W., Egea, I., Rossignol, M. et al. (2012) Proteomic analysis of chloroplast-to-chromoplast transition in tomato reveals metabolic shifts coupled with disrupted thylakoid biogenesis machinery and elevated energy-production components. *Plant Physiol.* 160, 708-725. doi:10.1104/pp.112.203679

Bermúdez, L., De Godoy, F., Baldet, P., Demarco, D., Osorio, S., Quadrana, L. et al. (2014) Silencing of the tomato Sugar Partitioning Affecting protein (SPA) modifies sink strength through a shift in leaf sugar metabolism. *Plant J.* 77, 676-687. doi:10.1111/tpj.12418

Bhuiyan, N.H., Rowland, E., Friso, G., Ponnala, L., Michel, E.J.S., and Van Wijk, K.J. (2020) Autocatalytic processing and substrate specificity of Arabidopsis chloroplast glutamyl peptidase. *Plant Physiol.* 184, 110-129. doi:10.1104/pp.20.00752

Cai, B., Li, Q., Xu, Y., Yang, L., Bi, H., and Ai, X. (2016) Genome-wide analysis of the fructose 1,6-bisphosphate aldolase (FBA) gene family and functional characterization of FBA7 in tomato. *Plant Physiol. Biochem.* 108, 251-265. doi:10.1016/j.plaphy.2016.07.019

Chen, S., Vaghchhipawala, Z., Li, W., Asard, H., and Dickman, M.B. (2004) Tomato phospholipid hydroperoxide glutathione peroxidase inhibits cell death induced by Bax and oxidative stresses in yeast and plants. *Plant Physiol.* 135, 1630-1641. doi:10.1104/pp.103.038091

Cheng, F., Zhou, Y.-H., Xia, X.-J., Shi, K., Zhou, J., and Yu, J.-Q. (2014) Chloroplastic thioredoxin-f and thioredoxin-m1/4 play important roles in brassinosteroids-induced changes in CO(2) assimilation and cellular redox homeostasis in tomato. *J. Expt. Bot.* 65, 4335-4347. doi:10.1093/jxb/eru207

D’Andrea, L., and Rodriguez-Concepcion, M. (2019) Manipulation of plastidial protein quality control components as a new strategy to improve carotenoid contents in tomato fruit. *Front. Plant Sci.* 10. doi:10.3389/fpls.2019.01071

Depège, N., Drevet, J., and Boyer, N. (1998) Molecular cloning and characterization of tomato cDNAs encoding glutathione peroxidase-like proteins. *Eur. J. Biochem.* 253, 445-451

Espinoza-Corral, R., Heinz, S., Klingl, A., Jahns, P., Lehmann, M., Meurer, J. et al. (2019) Plastoglobular protein 18 is involved in chloroplast function and thylakoid formation. *J. Expt. Bot.* 70, 3981-3993. doi:10.1093/jxb/erz177

Falara, V., Akhtar, T.A., Nguyen, T.T.H., Spyropoulou, E.A., Bleeker, P.M., Schauvinhold, I. et al. (2011) The tomato Terpene Synthase gene family. *Plant Physiol.* 157, 770-789. doi:10.1104/pp.111.179648

Gabruk, M., and Mysliwa-Kurdziel, B. (2020) The origin, evolution and diversification of multiple isoforms of light-dependent protochlorophyllide oxidoreductase (LPOR): focus on angiosperms. *Biochem. J*. 477, 2221-2236. doi:10.1042/bcj20200323

Gao, S., Gao, W., Liao, X., Xiong, C., Yu, G., Yang, Q. et al. (2019) The tomato *WV* gene encoding a thioredoxin protein is essential for chloroplast development at low temperature and high light intensity. *BMC Plant Biol.* 19, 265. doi:10.1186/s12870-019-1829-4

Giglione, C., Serero, A., Pierre, M., Boisson, B., and Meinnel, T. (2000) Identification of eukaryotic peptide deformylases reveals universality of N-terminal protein processing mechanisms. *EMBO J*. 19, 5916-5929. doi:10.1093/emboj/19.21.5916

Gu, Y.-Q., Chao, W.S., and Walling, L.L. (1996) Localization and post-translational processing of the wound-induced leucine aminopeptidase proteins of tomato. *J. Biol. Chem.* 271, 25880-25887. doi:10.1074/jbc.271.42.25880

Guo, Y., Huang, C., Xie, Y., Song, F., and Zhou, X. (2010) A tomato glutaredoxin gene SlGRX1 regulates plant responses to oxidative, drought and salt stresses. *Planta* 232, 1499-1509. doi:10.1007/s00425-010-1271-1

Heitz, T., Bergey, D.R., and Ryan, C.A. (1997) A gene encoding a chloroplast-targeted lipoxygenase in tomato leaves is transiently induced by wounding, systemin, and methyl jasmonate. *Plant Physiol.* 114, 1085-1093. doi:10.1104/pp.114.3.1085

Howe, G.A., Lee, G.I., Itoh, A., Li, L., and Derocher, A.E. (2000) Cytochrome P450-dependent metabolism of oxylipins in tomato. Cloning and expression of Allene Oxide Synthase and Fatty Acid Hydroperoxide Lyase. *Plant Physiol.* 123, 711-724. doi:10.1104/pp.123.2.711

Hu, Z., Lv, X., Xia, X., Zhou, J., Shi, K., Yu, J. et al. (2016) Genome-wide identification and expression analysis of Calcium-dependent Protein Kinase in tomato. *Front. Plant Sci.* 7, 469. doi:10.3389/fpls.2016.00469

Ishiga, Y., Ishiga, T., Wangdi, T., Mysore, K.S., and Uppalapati, S.R. (2012) NTRC and chloroplast-generated reactive oxygen species regulate *Pseudomonas syringae* pv. *tomato* disease development in tomato and *Arabidopsis*. *Mol. Plant Microbe Inter.* 25, 294-306. doi:10.1094/MPMI-05-11-0130

Islam, S., Rahman, I.A., Islam, T., and Ghosh, A. (2017) Genome-wide identification and expression analysis of glutathione S-transferase gene family in tomato: Gaining an insight to their physiological and stress-specific roles. *PLoS One* 12, e0187504. doi:10.1371/journal.pone.0187504

Jones, M.O., Perez-Fons, L., Robertson, F.P., Bramley, P.M., and Fraser, P.D. (2013) Functional characterization of long-chain prenyl diphosphate synthases from tomato. *Biochem. J.* 449, 729-740. doi:10.1042/bj20120988

Laizet, Y., Pontier, D., Mache, R., and Kuntz, M. (2004) Subfamily organization and phylogenetic origin of genes encoding plastid lipid-associated proteins of the fibrillin type *J. Genome Sci. Tech.* 3, 19-28

Laxalt, A.M., Ter Riet, B., Verdonk, J.C., Parigi, L., Tameling, W.I.L., Vossen, J. et al. (2003) Characterization of five tomato phospholipase D cDNAs: rapid and specific expression of LePLDβ1 on elicitation with xylanase. *Plant J.* 26, 237-247. doi:10.1046/j.1365-313x.2001.01023.x

Lee, J.-Y., Lee, H.-S., Song, J.-Y., Jung, Y.J., Reinbothe, S., Park, Y.-I. et al. (2013) Cell Growth Defect Factor1/CHAPERONE-LIKE PROTEIN OF POR1 plays a role in stabilization of light-dependent Protochlorophyllide Oxidoreductase in *Nicotiana benthamiana* and Arabidopsis. *Plant Cell* 25, 3944-3960. doi:10.1105/tpc.113.111096

Leitner-Dagan, Y., Ovadis, M., Zuker, A., Shklarman, E., Ohad, I., Tzfira, T. et al. (2006) CHRD, a plant member of the evolutionarily conserved YjgF family, influences photosynthesis and chromoplastogenesis. *Planta* 225, 89-102. doi:10.1007/s00425-006-0332-y

Lu, Y. (2016) Identification and roles of Photosystem II assembly, stability, and repair factors in Arabidopsis. *Front. Plant Sci.* 7, 168. doi:10.3389/fpls.2016.00168

Mariutto, M., Duby, F., Adam, A., Bureau, C., Fauconnier, M.-L., Ongena, M. et al. (2011) The elicitation of a systemic resistance by *Pseudomonas putida* BTP1 in tomato involves the stimulation of two lipoxygenase isoforms. *BMC Plant Biol.* 11, 29. doi:10.1186/1471-2229-11-29

Matsuba, Y., Nguyen, T.T.H., Wiegert, K., Falara, V., Gonzales-Vigil, E., Leong, B. et al. (2013) Evolution of a complex locus for terpene biosynthesis in *Solanum*. *Plant Cell* 25, 2022-2036. doi:10.1105/tpc.113.111013

Najami, N., Janda, T., Barriah, W., Kayam, G., Tal, M., Guy, M. et al. (2008) Ascorbate peroxidase gene family in tomato: its identification and characterization. *Mol. Genet. Genomics* 279, 171-182. doi:10.1007/s00438-007-0305-2

Newman, S.M., Eannetta, N.T., Yu, H., Prince, J.P., Carmen De Vicente, M., Tanksley, S.D. et al. (1993) Organisation of the tomato polyphenol oxidase gene family. *Plant Mol. Biol.* 21, 1035-1051. doi:10.1007/bf00023601

Ofori, P.A., Mizuno, A., Suzuki, M., Martinoia, E., Reuscher, S., Aoki, K. et al. (2018) Genome-wide analysis of ATP binding cassette (ABC) transporters in tomato. *PLoS One* 13, e0200854. doi:10.1371/journal.pone.0200854

Paetzold, H., Garms, S., Bartram, S., Wieczorek, J., Urós-Gracia, E.-M., Rodríguez-Concepción, M. et al. (2010) The isogene *1-Deoxy-D-Xylulose 5-Phosphate Synthase 2* controls isoprenoid profiles, precursor pathway allocation, and density of tomato trichomes. *Mol. Plant* 3, 904-916. doi:10.1093/mp/ssq032

Pankratov, I., McQuinn, R., Schwartz, J., Bar, E., Fei, Z., Lewinsohn, E. et al. (2016) Fruit carotenoid-deficient mutants in tomato reveal a function of the plastidial isopentenyl diphosphate isomerase (IDI1) in carotenoid biosynthesis. *Plant J.* 88, 82-94. doi:10.1111/tpj.13232

Park, S.Y., Scranton, M.A., Stajich, J.E., Yee, A., and Walling, L.L. (2017) Chlorophyte aspartyl aminopeptidases: Ancient origins, expanded families, new locations, and secondary functions. *PLoS One* 12: e0185492. https://doi.org/10.1371/journal.pone.0185492,

Peltier, J.B., Cai, Y., Sun, Q., Zabrouskov, V., Giacomelli, L., Rudella, A. et al. (2006) The oligomeric stromal proteome of *Arabidopsis thaliana* chloroplasts. *Mol. Cell Prot.* 5, 114-133. doi:10.1074/mcp.M500180-MCP200

Pichersky, E., Bernatzky, R., Tanksley, S.D., Breidenbach, R.B., Kausch, A.P., and Cashmore, A.R. (1985) Molecular characterization and genetic mapping of two clusters of genes encoding chlorophyll a/b-binding proteins in *Lycospersicum esculentum* (tomato). *Gene* 40, 247-258. doi:10.1016/0378-1119(85)90047-2

Pichersky, E., Brock, T.G., Nguyen, D., Hoffman, N.E., Piechulla, B., Tanksley, S.D. et al. (1989) A new member of the *CAB* gene family: structure, expression and chromosomal location of *Cab-8*, the tomato gene encoding the Type III chlorophyll a/b-binding polypeptide of photosystem I. *Plant Mol. Biol.* 12, 257-270. doi:10.1007/bf00043203

Pichersky, E., Subramaniam, R., Whites, M.J., Reid, J., Aebersold, R., and Green, B.R. (1991) Chlorophyll a/b binding (CAB) polypeptides of CP29, the internal chlorophyll a/b complex of PSII: characterization of the tomato gene encoding the 26 kDa (type 1) polypeptide, and evidence for a second CP29 polypeptide. *Mol. Gen. Genet.* 227, 277-284. doi:10.1007/bf00259681

Pulido, P., Perello, C., and Rodriguez-Concepcion, M. (2012) New Iinsights into plant isoprenoid metabolism. *Mol. Plant* 5, 964-967. doi:10.1093/mp/sss088

Rodríguez-Concepción, M., Ahumada, I., Diez-Juez, E., Sauret-Güeto, S., Lois, L.M., Gallego, F. et al. (2001) 1-Deoxy-d-xylulose 5-phosphate reductoisomerase and plastid isoprenoid biosynthesis during tomato fruit ripening. *Plant J.* 27, 213-222. doi:10.1046/j.1365-313x.2001.01089.x

Rohdich, F., Wungsintaweekul, J., Luttgen, H., Fischer, M., Eisenreich, W., Schuhr, C.A. et al. (2000) Biosynthesis of terpenoids: 4-Diphosphocytidyl-2-C-methyl-D-erythritol kinase from tomato. *Proc. Natl. Acad. Sci. USA* 97, 8251-8256. doi:10.1073/pnas.140209197

Sade, D., Eybishtz, A., Gorovits, R., Sobol, I., and Czosnek, H. (2012) A developmentally regulated lipocalin-like gene is overexpressed in *Tomato yellow leaf curl virus*-resistant tomato plants upon virus inoculation, and its silencing abolishes resistance. *Plant Mol. Biol.* 80, 273-287. doi:10.1007/s11103-012-9946-6

Schilmiller, A.L., Schauvinhold, I., Larson, M., Xu, R., Charbonneau, A.L., Schmidt, A. et al. (2009) Monoterpenes in the glandular trichomes of tomato are synthesized from a neryl diphosphate precursor rather than geranyl diphosphate. *Proc.* *Natl. Acad. Sci. USA* 106, 10865-10870. doi:10.1073/pnas.0904113106

Schmid, J., Schaller, A., Leibinger, U., Boll, W., and Amrhein, N. (1992) The *in-vitro* synthesized tomato shikimate kinase precursor is enzymatically active and is imported and processed to the mature enzyme by chloroplasts. *Plant J.* 2, 375-383. doi:10.1046/j.1365-313x.1992.t01-36-00999.x

Schubert, M., Petersson, U.A., Haas, B.J., Funk, C., Schroder, W.P., and Kieselbach, T. (2002) Proteome map of the chloroplast lumen of *Arabidopsis thaliana*. *J. Biol. Chem.* 277, 8354-8365. doi:10.1074/jbc.M108575200

Schwartz, E., Shen, D., Aebersold, R., McGrath, J.M., Pichersky, E., and Green, B.R. (1991) Nucleotide sequence and chromosomal location of *Cab11* and *Cab12*, the genes for the fourth polypeptide of the photosystem I light-harvesting antenna (LHCI). *FEBS Lett.* 280, 229-234. doi:10.1016/0014-5793(91)80299-i

Sun, H., Fan, H.-J., and Ling, H.-Q. (2015) Genome-wide identification and characterization of the bHLH gene family in tomato. *BMC Genomics* 16, 9. doi:10.1186/s12864-014-1209-2

Tu, C.J., Park, S.Y., and Walling, L.L. (2003) Isolation and characterization of the neutral leucine aminopeptidase (*LapN*) of tomato. *Plant Physiol.* 132, 243-255

Vu, N.T., Kamiya, K., Fukushima, A., Hao, S., Ning, W., Ariizumi, T. et al. (2019) Comparative co-expression network analysis extracts the *SlHSP70* gene affecting to shoot elongation of tomato*. Plant Biotech.* 36, 143-153. doi:10.5511/plantbiotechnology.19.0603a

Wahyudi, A., Ariyani, D., Ma, G., Inaba, R., Fukasawa, C., Nakano, R. et al. (2018) Functional analyses of lipocalin proteins in tomato. *Plant Biotech.* 35, 303-312. doi:10.5511/plantbiotechnology.18.0620a

Wallbraun, M., Kim, S., Green, B.R., Piechulla, B., and Pichersky, E. (1994) Nucleotide sequence of a tomato *psbS* Gene. *Plant Physiol.* 106, 1703-1704. doi:10.1104/pp.106.4.1703

Walling, L.L. (2006) Recycling or regulation? The role of amino-terminal modifying enzymes. *Curr. Opin. Plant Biol.* 9, 227-233. doi: 10.1016/j.pbi.2006.03.009

Wang, L., Leister, D., Guan, L., Zheng, Y., Schneider, K., Lehmann, M. et al. (2020) The Arabidopsis SAFEGUARD1 suppresses singlet oxygen-induced stress responses by protecting grana margins. *Proc. Natl. Acad. Sci. USA* 117, 6918-6927. doi:10.1073/pnas.1918640117

Waseem, M., Ahmad, F., Habib, S., Gao, Y., and Li, Z. (2018) Genome-wide identification of FK506-binding domain protein gene family, its characterization, and expression analysis in tomato (*Solanum lycopersicum* L.). *Gene* 678, 143-154. doi:10.1016/j.gene.2018.08.021

Yamauchi, Y., Ejiri, Y., Toyoda, Y., and Tanaka, K. (2003) Identification and biochemical characterization of plant acylamino acid-releasing enzyme. *J. Biochem.* 134, 251-257. doi:10.1093/jb/mvg138

Yu, G., Nguyen, T.T.H., Guo, Y., Schauvinhold, I., Auldridge, M.E., Bhuiyan, N. et al. (2010) Enzymatic functions of wild tomato Methylketone Synthases 1 and 2. *Plant Physiol.* 154, 67-77. doi:10.1104/pp.110.157073
